# Supplementary material for: Transcriptional pausing at the translation start site operates as a critical checkpoint for riboswitch regulation
Source: Nat Commun. 2017 Jan 10;8:13892. doi: 10.1038/ncomms13892 (PMC5234074; doi:10.1038/ncomms13892)
Supplement: Supplementary Information — Supplementary Figures, Supplementary Tables and Supplementary References. [file ncomms13892-s1.pdf]

Supplementary Information

Supplementary Figures

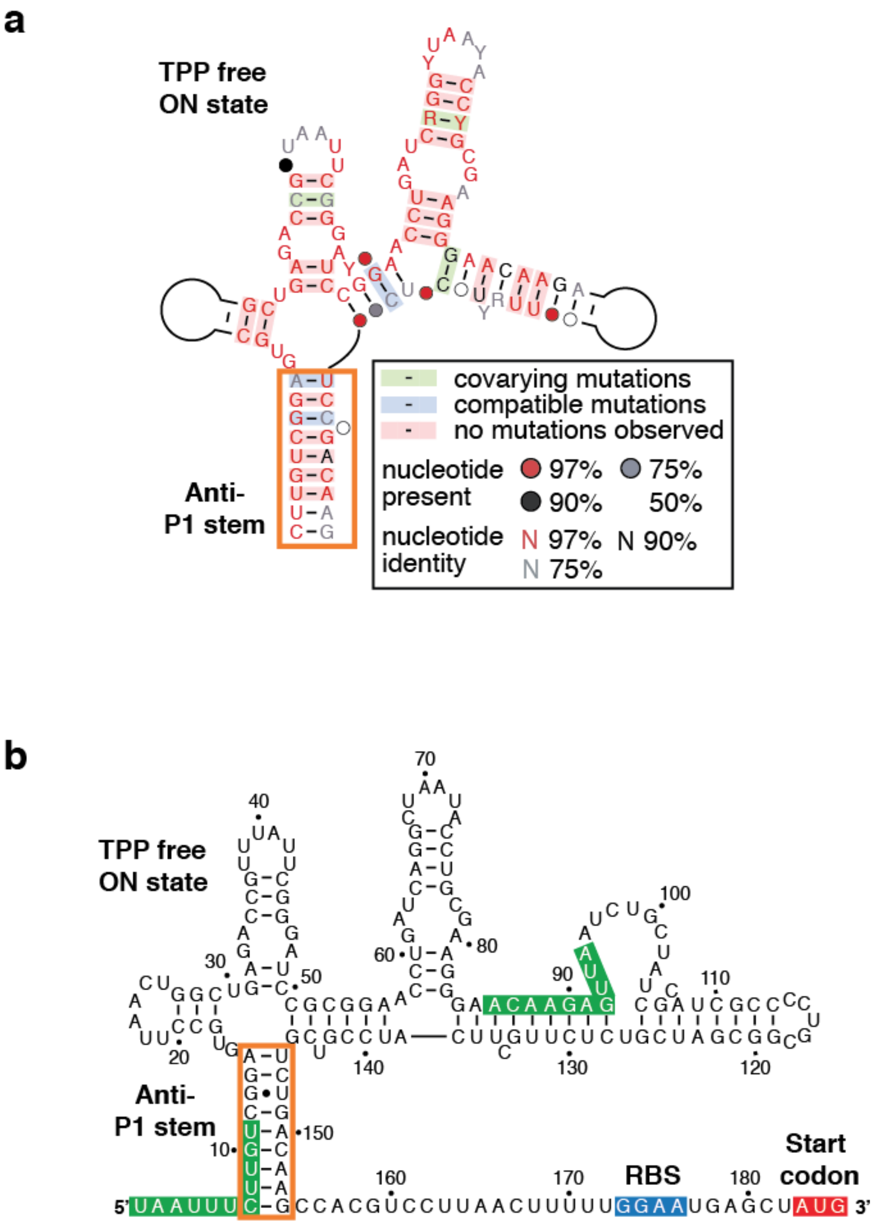

Supplementary Figure 1. Bioinformatic analysis of the *thiC* riboswitch.

(a) Consensus secondary structure obtained from an alignment of 77 different sequences of *thiC* riboswitches. The legend in the figure describes the color coding and the schematic loops represent variable-length stem loops. Nucleotides conserved at least at

97%, 90% or 75% are represented in red, black or gray, respectively. Bases for which the identity is not conserved but that are present in 97%, 90%, 75% or 50% of candidates are indicated by circles in red, black, gray or white, respectively. Base pairs that show conservation, covariation or compatibility (e.g., G-C to G-U) are shown in red, green or blue, respectively. The figure was drawn with R2R[1] from the Stockholm alignment in **Supplementary Data** and was refined to keep as many sequences as possible to best describe the structure corresponding to the *E. coli thiC* riboswitch.

(b) Secondary structure of the *thiC* riboswitch representing the TPP-free state. Sequences involved in the P1 stem, RBS and start codon are shown in green, blue and red, respectively. The anti-P1 stem is shown in an orange rectangle.

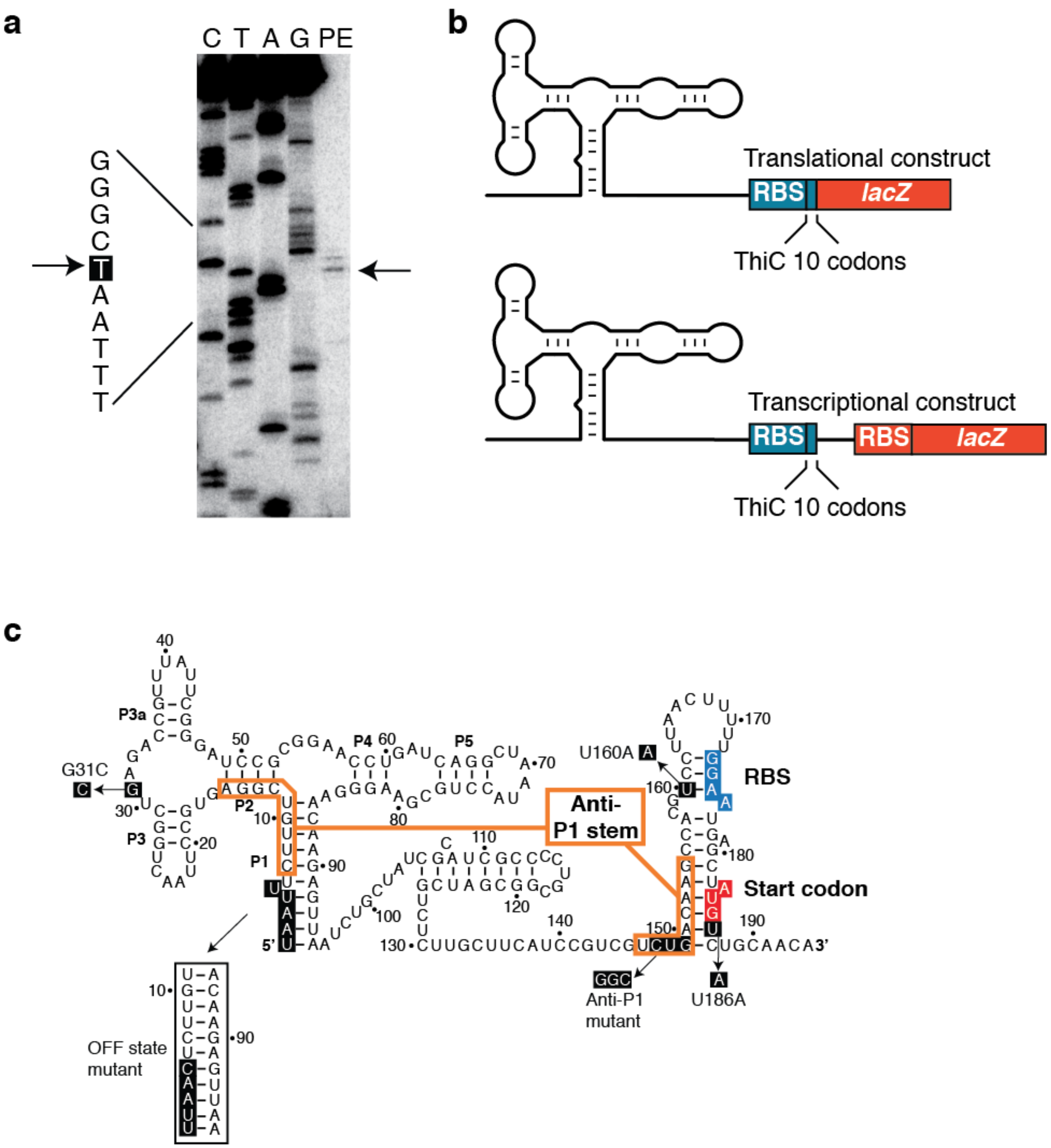

Supplementary Figure 2. Constructs of the *thiC* riboswitch used in this study.

(a) Determination of *thiC* transcription start site by primer extension. Total RNA extracted from *E. coli* was used with oligonucleotide AD5. The arrow represents the +1 transcription start site.

(b) Schematic of the *thiC* translational (top) and transcriptional (bottom) fusions. Both fusions contain an arabinose inducible promoter fused to the *thiC* riboswitch domain with

the first 10 *thiC* codons. While the translational fusion reports on both the mRNA and protein levels, the presence of an additional RBS site in the *lacZ* transcriptional fusion allows only to report the level of mRNA.

(c) Secondary structure of the *thiC* riboswitch and mutants used in this study. Mutations are shown in black. The OFF state mutant is expected to stabilize the P1 stem by allowing perfect base pairing between residues 1-5 and 92-96. The anti-P1 stem is designed to disrupt the formation of the anti-P1 stem when the RNA polymerase is stalled at the C187 pause site. The anti-P1 stem, the RBS and the start codon are shown in orange, blue and red, respectively.

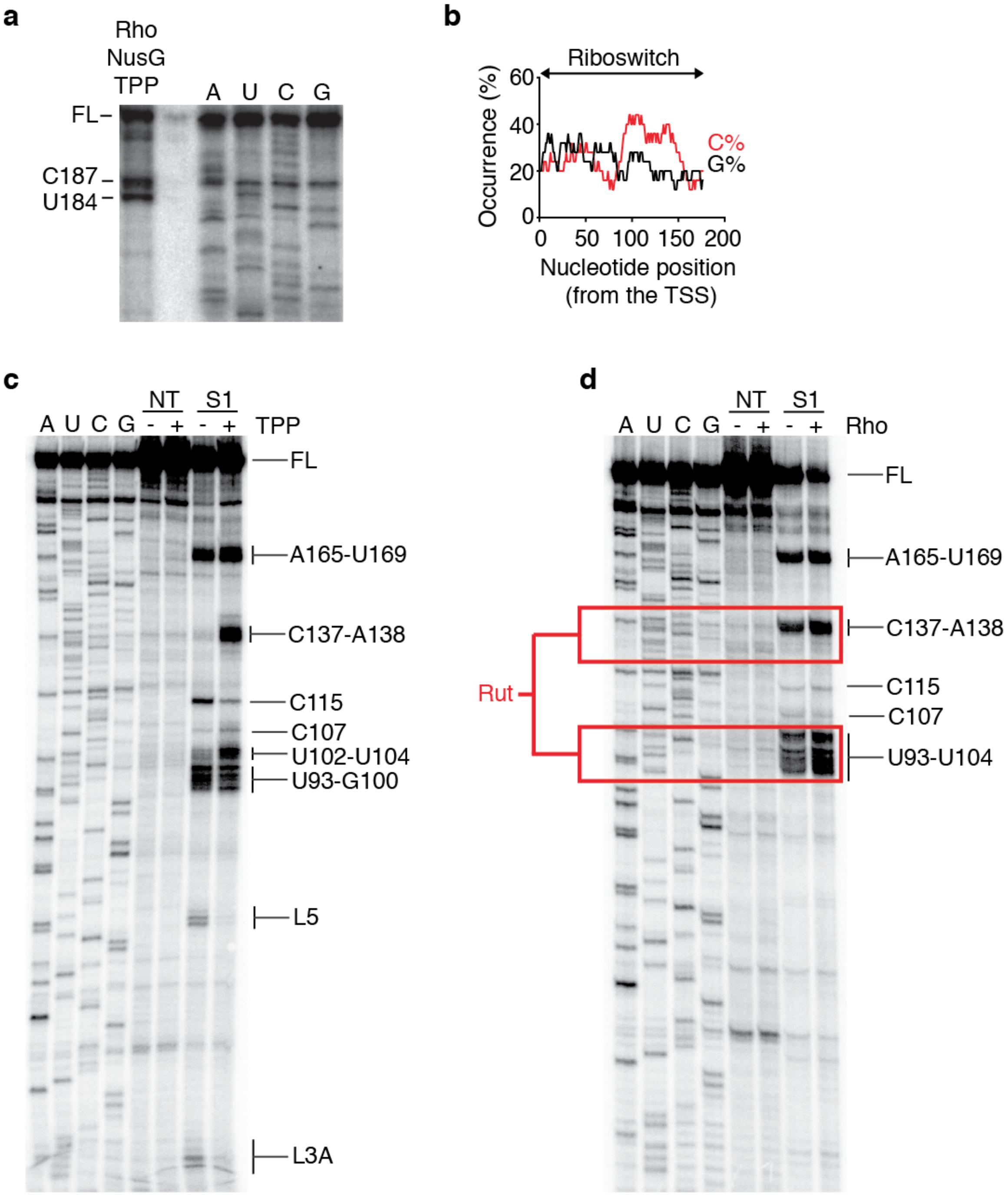

Supplementary Figure 3. Rho transcription termination of the *thiC* riboswitch.

(a) *In vitro* Rho-dependent transcription performed using the *thiC* riboswitch in the presence of 50 nM Rho, 50 nM NusG and 10 μM TPP. The determination of transcription

termination sites were determined using 3'-O-Methyl NTPs in the transcription elongation mix.

(b) Sequence analysis of cytosine (C%) and guanine (G%) distribution of the *thiC* riboswitch sequence. A scanning window of 25 nt was used to find the occurrences of G and C as a function or the transcription start site.

(c) Nuclease S1 probing of the *thiC* riboswitch in the absence (-) or presence (+) of 10 μM TPP. Reactions were done in the absence (NT) or presence (S1) of nuclease S1. Regions showing significant cleavage changes are indicated on the right. The determination of S1 cleavage sites sites was performed using 3'-O-Methyl NTPs in the transcription elongation mix.

(d) Nuclease S1 probing of the *thiC* riboswitch performed in the presence of 10 μM TPP. Reactions were done in the absence (-) or presence (+) of 1 μM Rho and in the absence (NT) or presence (S1) of nuclease S1. Regions showing significant cleavage changes are indicated on the right. The predicted rut site is indicated by red boxes. The determination of S1 cleavage sites sites was performed using 3'-O-Methyl NTPs in the transcription elongation mix.

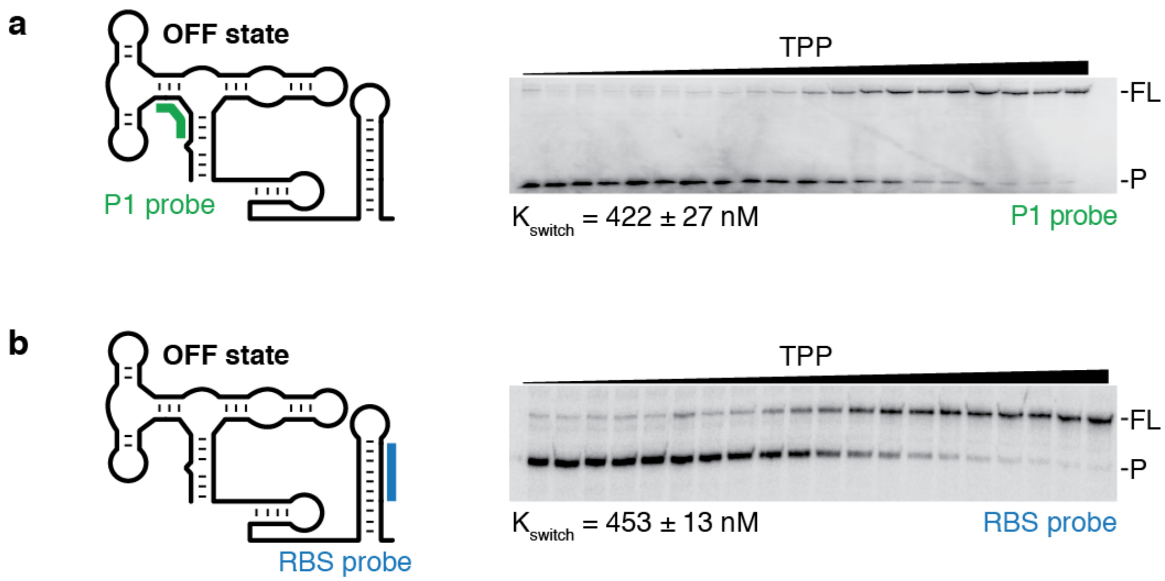

Supplementary Figure 4. K_{switch} determination of the *thiC* riboswitch using P1 or RBS probe.

(a) K_{switch} determination using the P1 probe done in the presence of increasing TPP concentrations ranging from 100 pM to 100 μM. A K_{switch} value of 422 ± 27 nM was obtained. The targeted region by the P1 probe is indicated on the cartoon to the left. Full length (FL) and cleaved products (P) are indicated on the right.

(b) K_{switch} determination using the RBS probe done in the presence of increasing TPP concentrations ranging from 100 pM to 100 μM. A K_{switch} value of 453 nM ± 13 nM was obtained. The targeted region by the RBS probe is indicated on the cartoon to the left. Full length (FL) and cleaved products (P) are indicated on the right.

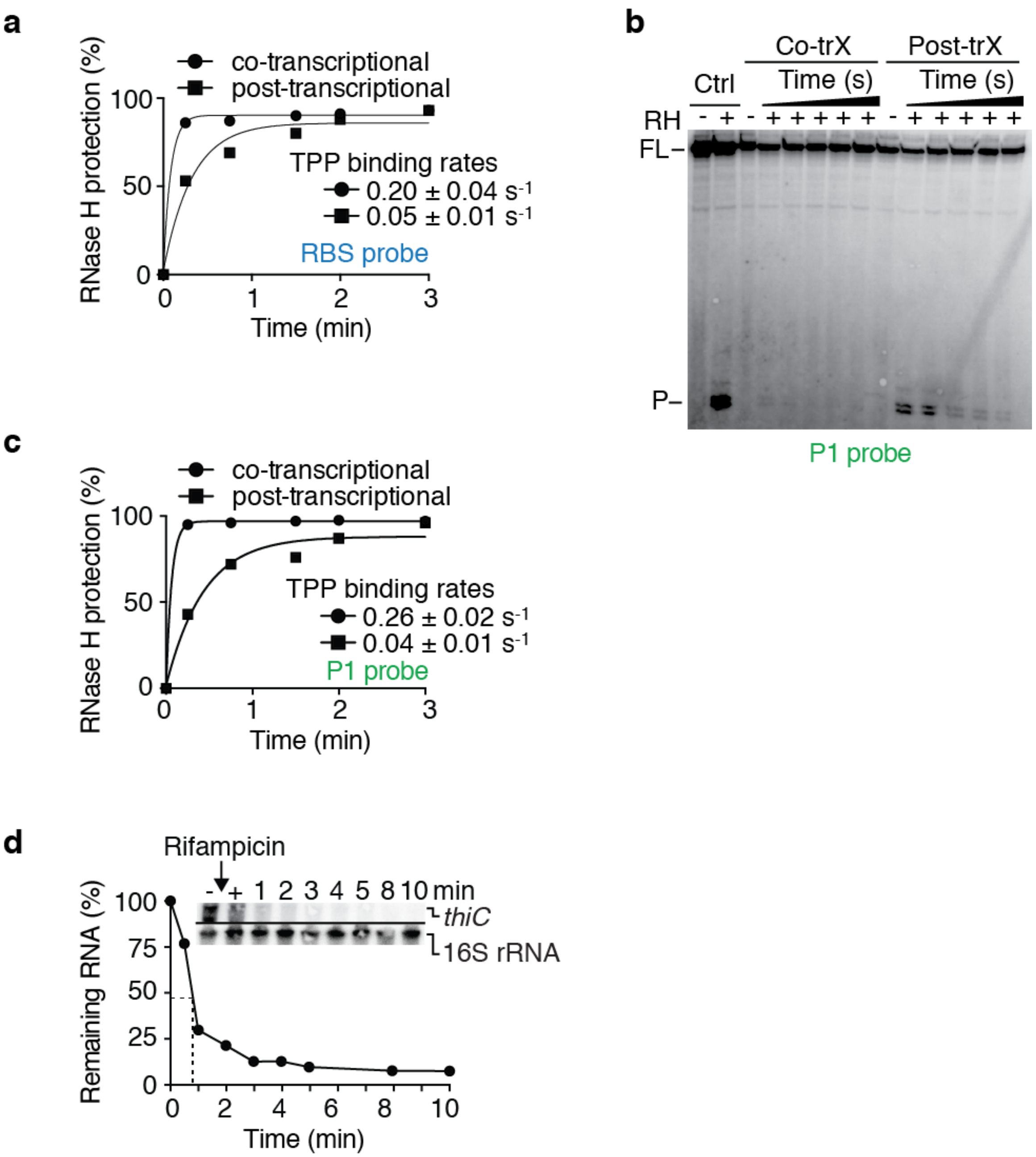

Supplementary Figure 5. Kinetics of TPP binding to the *thiC* riboswitch and half-life of the *thiC* transcript *in vivo*.

(a) Quantification analysis of RNase H experiments using the RBS probe. Data were fitted to a single-exponential model and yielded apparent TPP binding rates of 0.20 s^{-1} ±

0.04 s^{-1} and 0.05 s^{-1} ± 0.01 s^{-1} for cotranscriptional and post-transcriptional binding, respectively.

(b) RNase H probing assays monitoring TPP binding using the P1 probe. Control reactions (Ctrl) done in the absence (-) or presence (+) of RNase H (RH) indicate the presence of cleaved products (P) in the absence of TPP. Post-transcriptional and cotranscriptional reactions were done as described in **Fig. 2c**. The full length (FL) and cleaved product (P) are indicated on the left.

(c) Quantification analysis of RNase H experiments using the P1 probe. Data were fitted to a single-exponential model and yielded apparent TPP binding rates of 0.26 s^{-1} ± 0.02 s^{-1} and 0.04 s^{-1} ± 0.01 s^{-1} for cotranscriptional and post-transcriptional binding, respectively.

(d) Northern blot assays performed as a function of time using the *E. coli* MG1665 strain[2] at various time points before (-) and after (+) rifampicin (250 μg per mL) addition. The half-life of the mRNA was deduced from the densitometry analysis, which was estimated to be < 60 s.

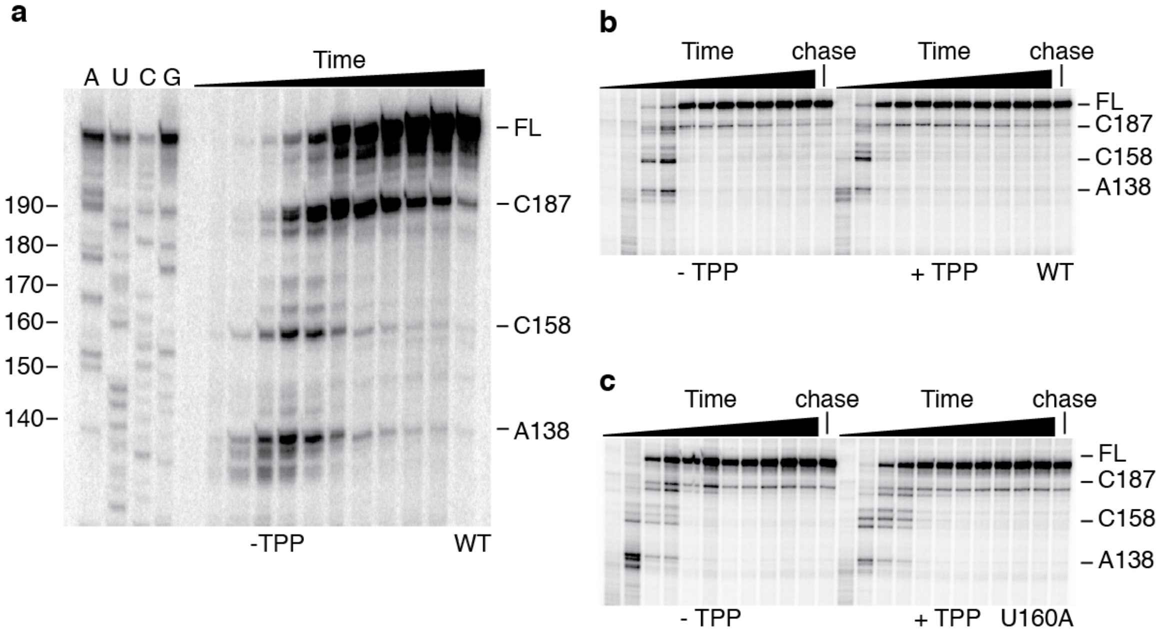

Supplementary Figure 6. Transcriptional pausing of the wild type *thiC* riboswitch and selected mutants.

(a) Mapping of *thiC* riboswitch transcriptional intermediates done in the absence of TPP. Transcription reactions were performed using 25 μM NTPs. Pause sites were mapped by using 3'-O-Methyl NTPs in the transcription elongation mix. Transcriptional intermediates are indicated on the right.

(b) Transcriptional pausing of the wild type *thiC* riboswitch in the absence or presence of 10 μM TPP. Reactions were done in the presence of 100 μM NTPs and were incubated for 15 s, 30 s, 45 s, 60 s, 90 s, 2 min, 3 min, 4 min, 5 min, 8 min and 10 min. Chase reactions were done by adding 1 mM NTPs.

(c) Transcriptional pausing of the U160A riboswitch mutant in the absence or presence of 10 μM TPP. Reactions were done in the presence of 100 μM NTPs and were incubated for 15 s, 30 s, 45 s, 60 s, 90 s, 2 min, 3 min, 4 min, 5 min, 8 min and 10 min. Chase reactions were done by adding 1 mM NTPs.

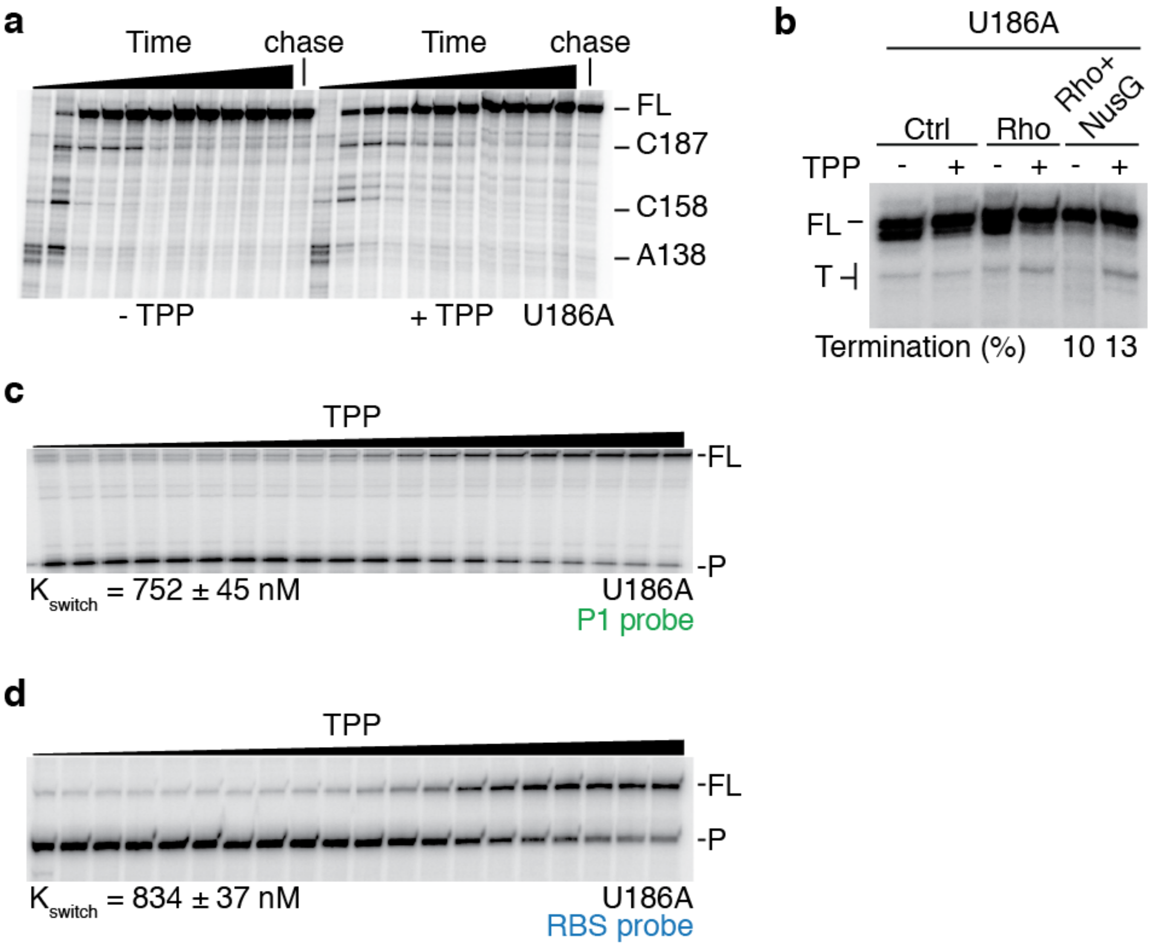

Supplementary Figure 7. The U186A riboswitch mutant strongly inhibits Rho transcription termination.

(a) Transcriptional pausing of the U186A riboswitch mutant in the absence or presence of 10 μM TPP. Reactions were done in the presence of 100 μM NTPs and were incubated for 15 s, 30 s, 45 s, 60 s, 90 s, 2 min, 3 min, 4 min, 5 min, 8 min and 10 min. Chase reactions were done by adding 1 mM NTPs.

(b) *In vitro* Rho-dependent transcription performed using the U186A riboswitch mutant in the absence (-) or presence (+) of 10 μM TPP. Reactions were done in the absence (Ctrl) or presence of 50 nM Rho or 50 nM NusG

(c) K_{switch} determination of the U186A mutant using the P1 probe done in the presence of increasing TPP concentrations ranging from 100 pM to 100 μM. A K_{switch} value of 752 nM ± 45 nM was obtained. Full length (FL) and cleaved product (P) are indicated on the right.

(d) K_{switch} determination of the U186A mutant using the RBS probe done in the presence of increasing TPP concentrations ranging from 100 pM to 100 μM. A K_{switch} value of 834 nM ± 37 nM was obtained. Full length (FL) and cleaved product (P) are indicated on the right.

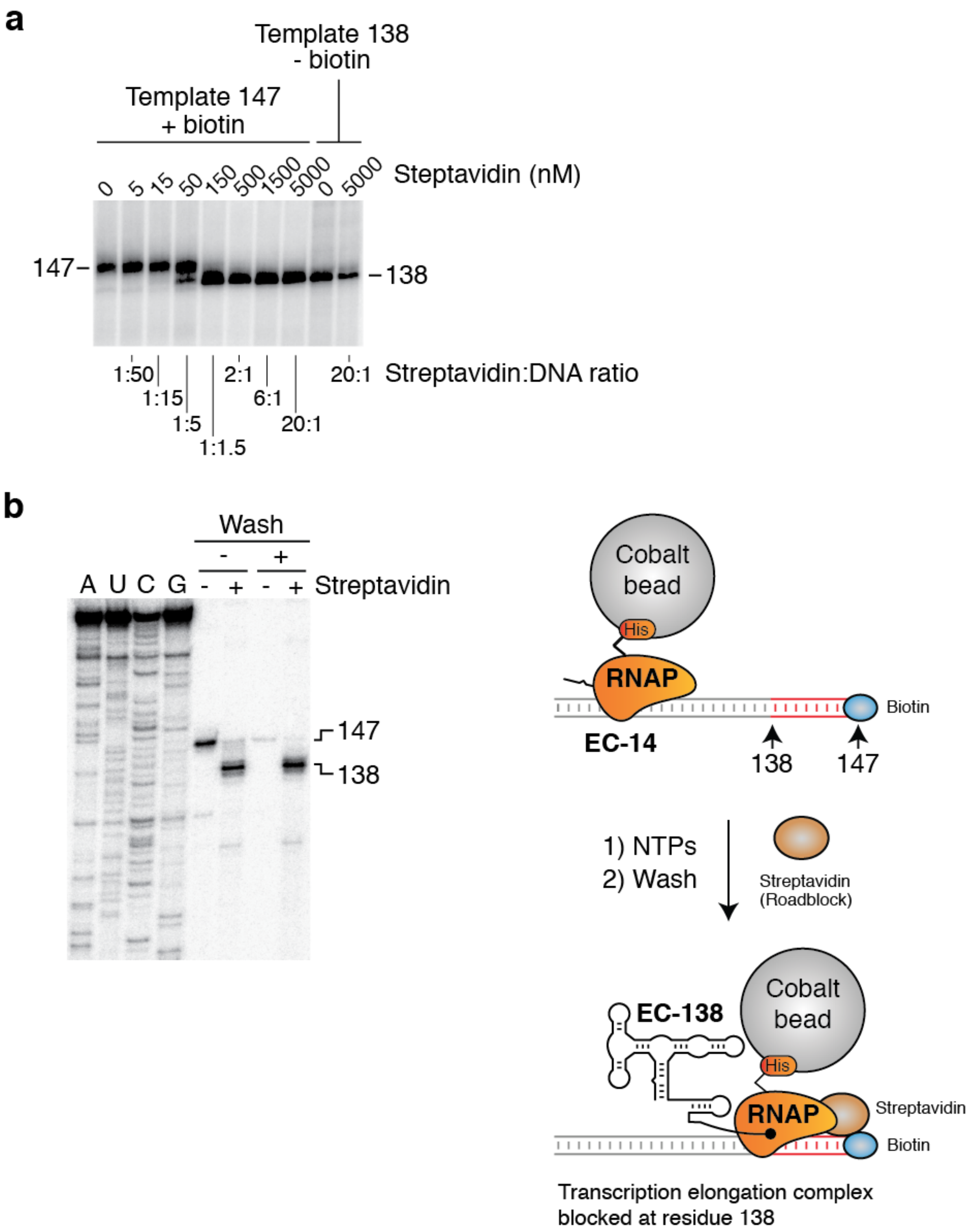

Supplementary Figure 8. Formation of stable transcription elongation complexes using a biotin-streptavidin roadblock.

(a) Transcription reactions performed as a function of streptavidin concentration. In the absence of streptavidin, a transcript of 147 nt is observed. However, the addition of streptavidin results in the appearance of a 138 nt transcript due to the formation of the biotin-streptavidin roadblock. A template allowing the production of a 138 nt transcript is used as a molecular weight marker. The ratio of streptavidin:DNA is shown at the bottom. **(b)** Control experiments showing the integrity of transcription elongation complexes stalled at a biotin-streptavidin roadblock. In these experiments, cobalt beads were used as solid support to pull down transcription complexes. The schematic describes the experimental procedure in which the addition of NTPs followed by a washing step yield transcription complexes stalled at position 138. A representative gel shows that the 138 nt transcript is retained after a washing step only in the presence of streptavidin, consistent with the presence of intact elongation complexes. In the absence of a washing step, both 147 nt and 138 nt transcripts are obtained in the absence or presence of streptavidin, respectively.

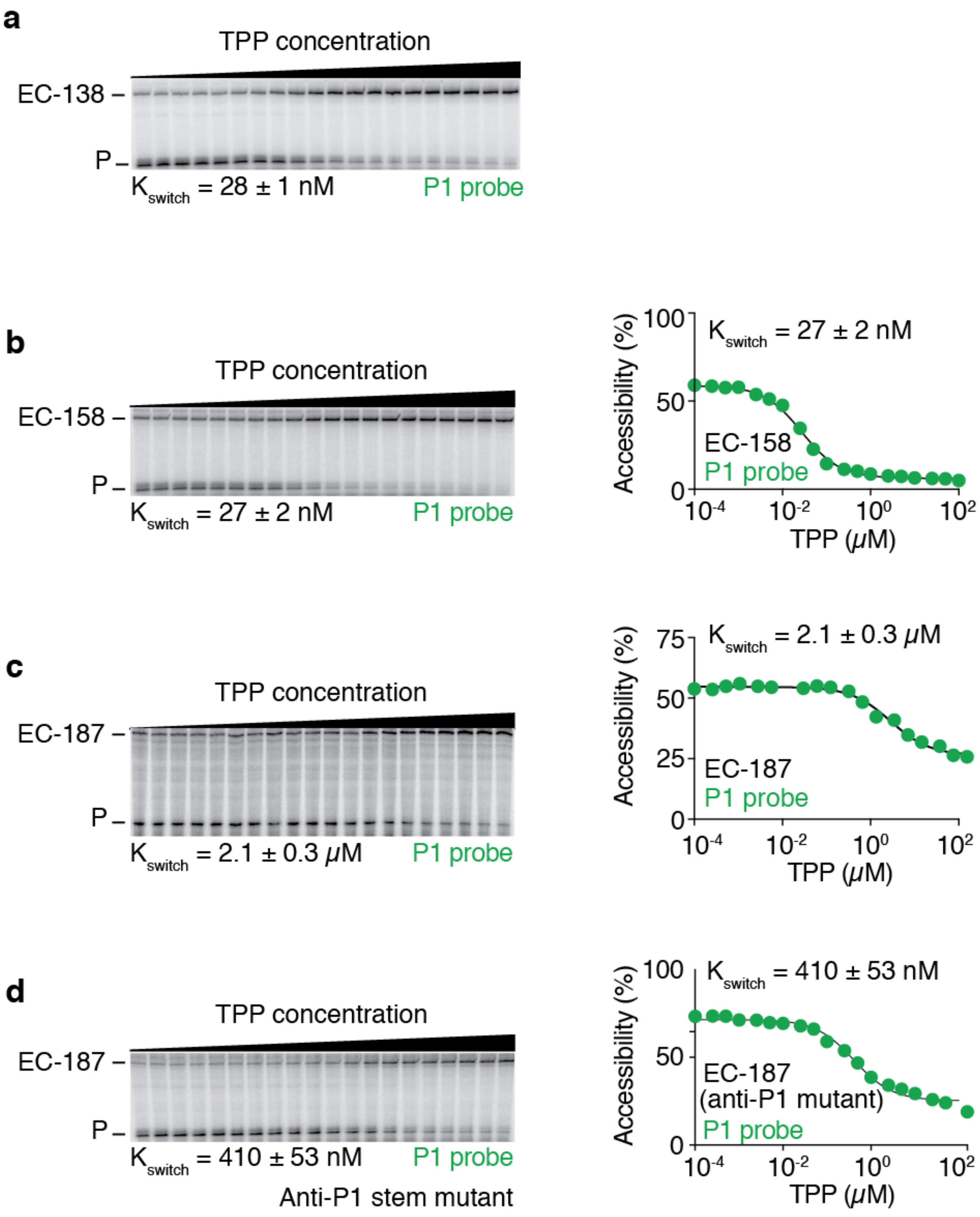

Supplementary Figure 9. Probing *thiC* riboswitch elongation complexes stalled at transcriptional pause sites.

(a) K_{switch} determination of EC-138. Reactions were done as a function of TPP concentration ranging from 100 pM to 100 μM. A K_{switch} value of 28 nM ± 1 nM was obtained using the P1 probe. The quantification is shown in **Fig 4c**.

(b) K_{switch} determination of EC-158. Reactions were done as a function of TPP concentration ranging from 100 pM to 100 μM using the P1 probe. A quantification of the data is shown on the right in which a K_{switch} value of 27 nM ± 2 nM was obtained.

(c) K_{switch} determination of EC-187. Reactions were done as a function of TPP concentration ranging from 100 pM to 100 μM using the P1 probe. A quantification of the data is shown on the right in which a K_{switch} value of 2.1 μM ± 0.43 μM was obtained.

(d) K_{switch} determination of EC-187 in the context of the anti-P1 stem mutant (C147G/U148G/G149C). Reactions were done as a function of TPP concentration ranging from 100 pM to 100 μM using the P1 probe. A quantification of the data is shown on the right in which a K_{switch} value of 410 nM ± 53 nM was obtained.

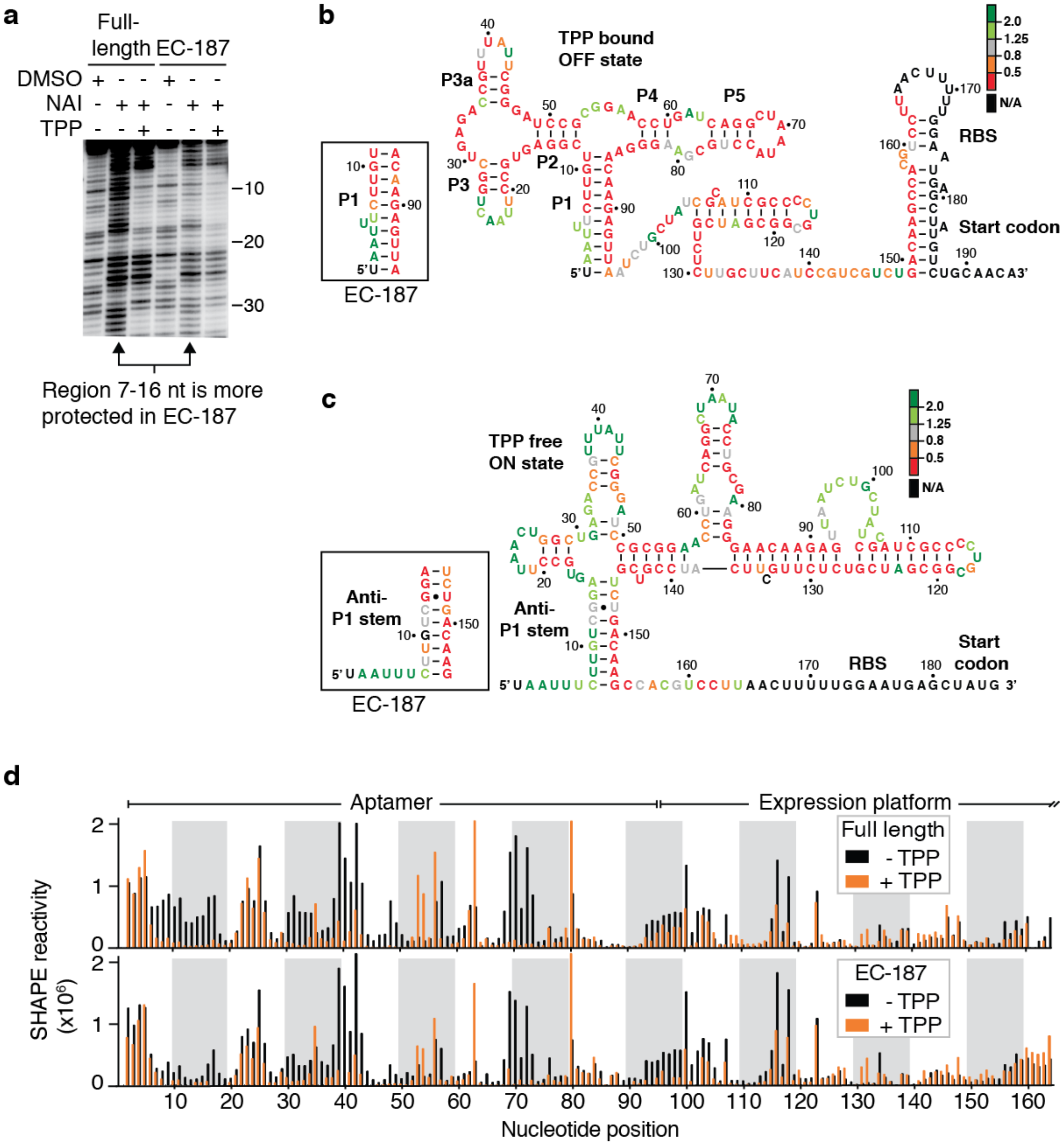

Supplementary Figure 10. SHAPE probing of *thiC* riboswitch elongation complexes stalled at transcriptional pause sites.

(a) SHAPE modifications of the *thiC* riboswitch full-length sequence or in the context of the EC-187 elongation complex performed in the absence (-) or presence (+) of 10 μM TPP and in the absence (-) or presence of 150 mM NAI. Nucleotide positions are indicated on the right. Control experiments were done in the presence of DMSO.

(b) Secondary structure of the *thiC* riboswitch OFF state showing the summary of NAI protection obtained in the presence of 10 μM TPP. SHAPE results for the EC-187 construct are shown in insert. Nucleotides for which no information could be obtained are indicated in black (N/A).

(c) Secondary structure of the *thiC* riboswitch ON state showing the summary of NAI protection obtained in the absence of TPP. SHAPE results for the EC-187 construct are shown in insert. Nucleotides for which no information could be obtained are indicated in black (N/A).

(d) SHAPE modification of the *thiC* riboswitch performed on the full-length transcript and EC-187 elongation complex in the absence and presence of 10 μM TPP. The histogram represents the relative SHAPE reactivity for positions 1 to 164.

a

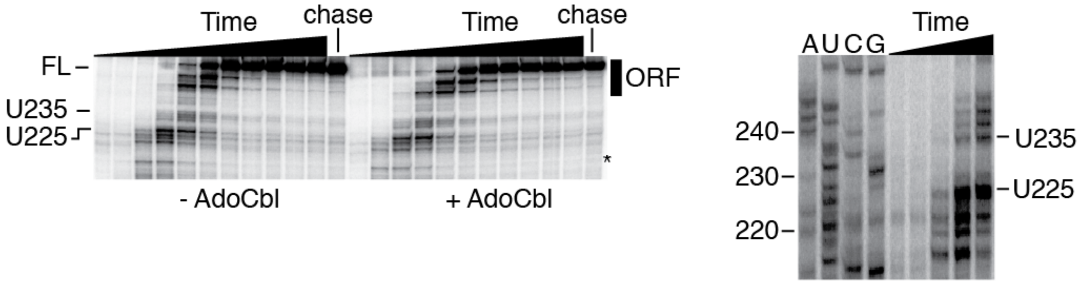

b

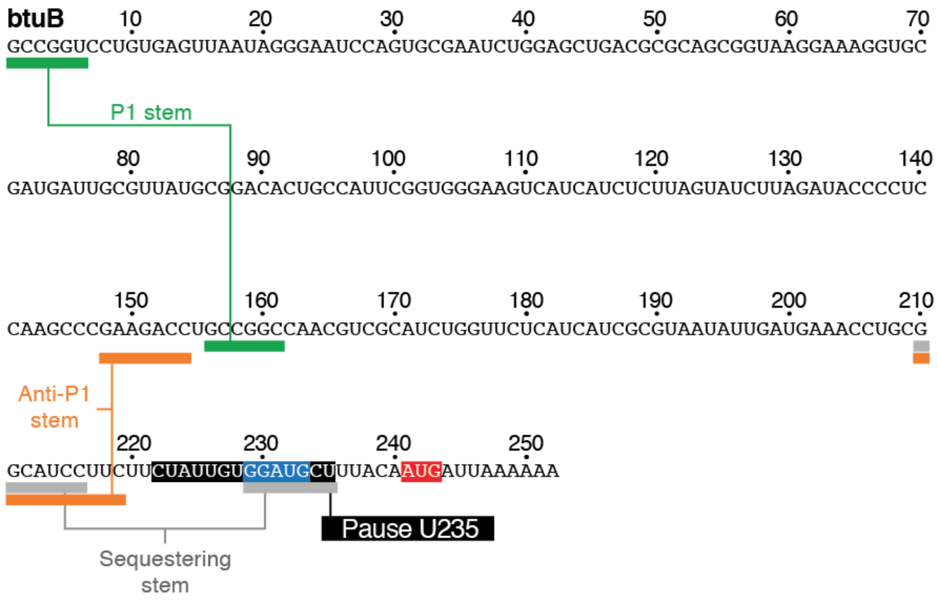

Supplementary Figure 11. Transcriptional pausing of the *btuB* riboswitch.

(a) Transcriptional pausing of the *btuB* riboswitch in the absence or presence of 100 μM AdoCbl. Reactions were done in the presence of 25 μM NTPs and were incubated for 15 s, 30 s, 45 s, 60 s, 90 s, 2 min, 3 min, 4 min, 5 min, 8 min and 10 min. Chase reactions were done by adding 1 mM NTPs. Transcriptional intermediates are indicated on the right. Transcriptional pause sites found downstream in the ORF or exhibiting low efficiency (shown by a star) were not taken into account. The right panel shows the pause

site mapping of *btuB* transcript intermediates. Reaction times were performed for 15 s, 30 s, 45 s, 60 s and 90 s.

(b) Sequence of the *btuB* riboswitch showing the predicted P1, anti-P1 and sequestering stems are indicated in green, orange and gray, respectively. The expected region sequestered by RNAP at the U235 pause site is shown in black. The prediction of paired regions is based on a previous study[3].

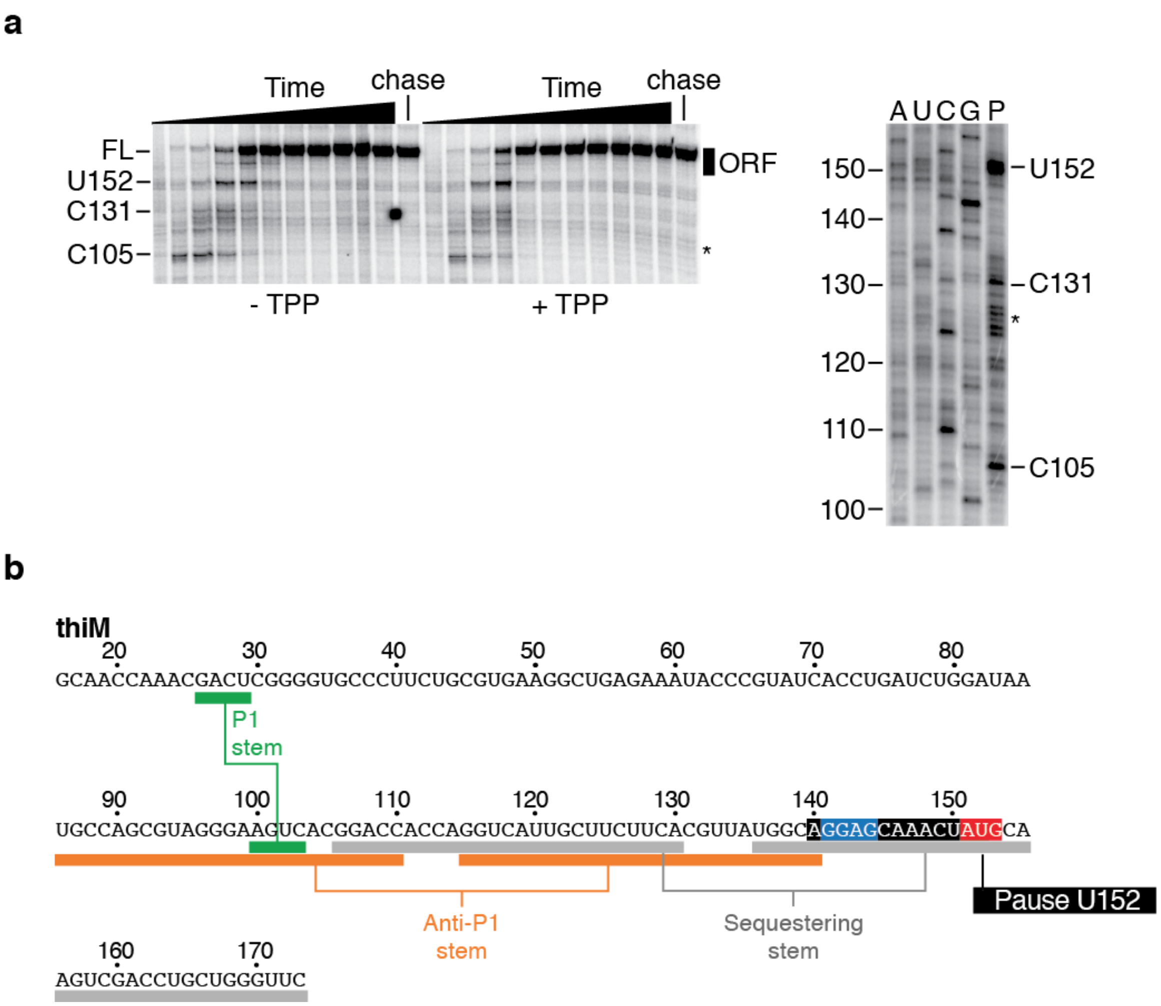

Supplementary Figure 12. Transcriptional pausing of the *thiM* riboswitch.

(a) Transcriptional pausing of the *thiM* riboswitch in the absence or presence of 10 μM TPP. Reactions were done in the presence of 25 μM NTPs and were incubated for 15 s, 30 s, 45 s, 60 s, 90 s, 2 min, 3 min, 4 min, 5 min, 8 min and 10 min. Chase reactions were done by adding 1 mM NTPs. Transcriptional intermediates are indicated on the right. Transcriptional pause sites found downstream in the ORF or exhibiting low efficiency (shown by a star) were not taken into account. The right panel shows the pause site mapping of *thiM* transcript intermediates. A reaction time (P) was performed at 45 s.

(b) Sequence of the *thiM* riboswitch showing the predicted P1, anti-P1 and sequestering stems are indicated in green, orange and gray, respectively. The expected region sequestered by RNAP at the U152 pause site is shown in black. The prediction of paired regions is based on a previous study[4].

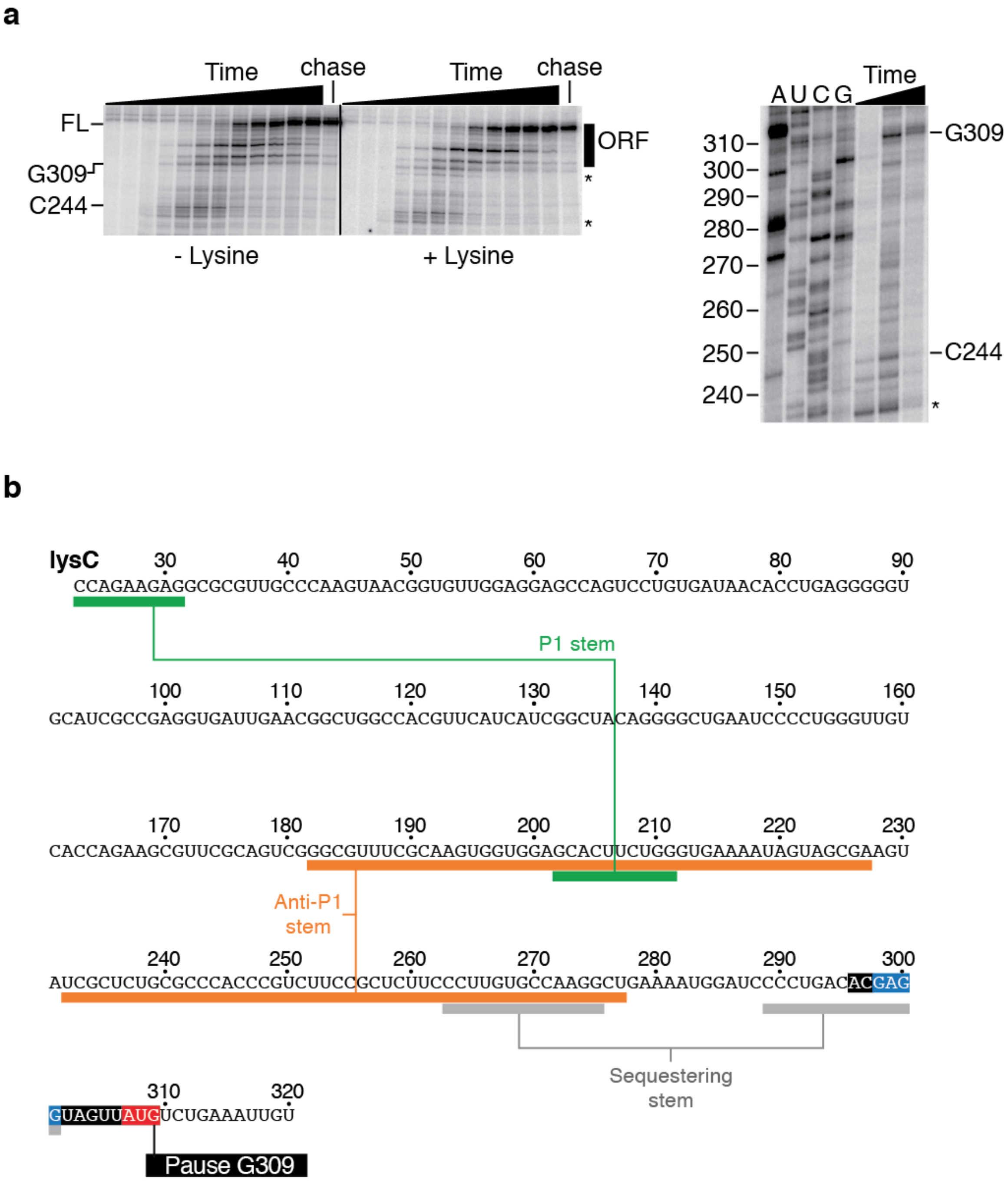

Supplementary Figure 13. Transcriptional pausing of the *lysC* riboswitch.

(a) Transcriptional pausing of the *lysC* riboswitch in the absence or presence of 1 mM lysine. Reactions were done in the presence of 50 μM NTPs and were incubated for 10 s,

30 s, 45 s, 60 s, 90 s, 120 s, 150 s, 3 min, 4 min, 5 min, 6 min, 8 min and 10 min. Chase reactions were done by adding 1 mM NTPs. Transcriptional intermediates are indicated on the right. Transcriptional pause sites found downstream in the ORF or exhibiting low efficiency (shown by a star) were not taken into account. The right panel shows the pause site mapping of *lysC* transcript intermediates. Reaction times were performed for 60 s, 90 s and 120 s.

(b) Sequence of the *lysC* riboswitch showing the predicted P1, anti-P1 and sequestering stems are indicated in green, orange and gray, respectively. The expected region sequestered by RNAP at the G304 pause site is shown in black. The prediction of paired regions is based on a previous study[5].

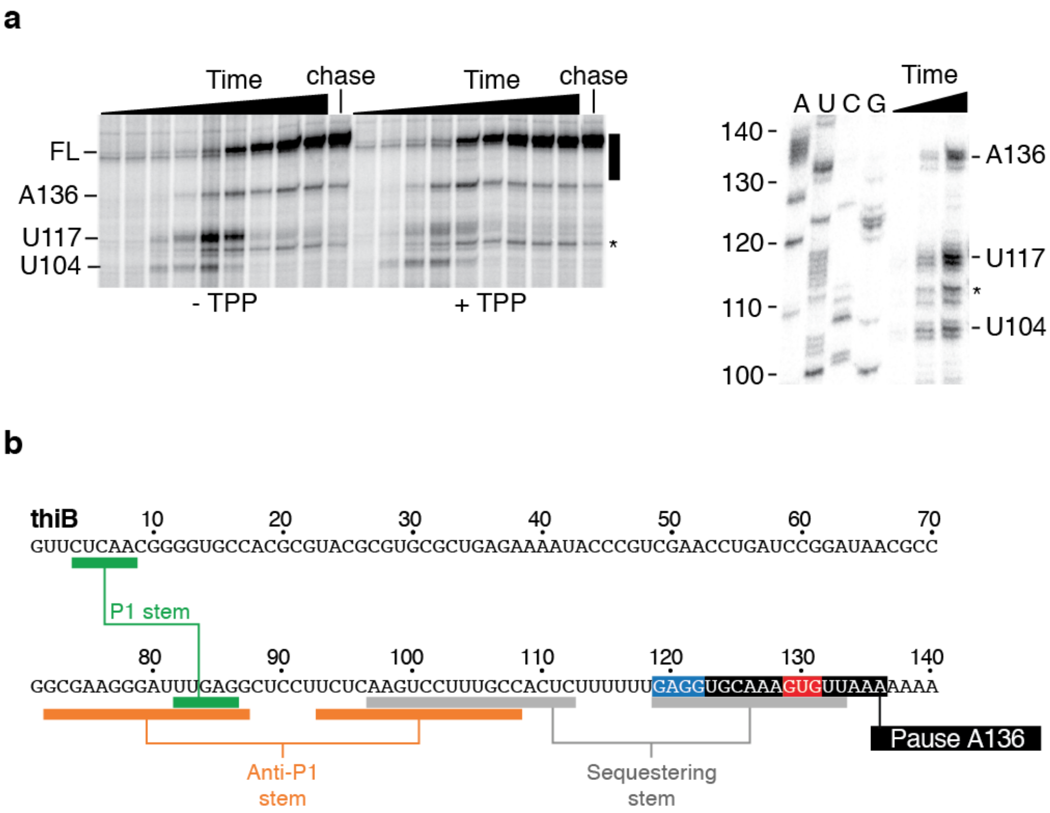

Supplementary Figure 14. Transcriptional pausing of the *thiB* riboswitch.

(a) Transcriptional pausing of the *thiB* riboswitch in the absence or presence of 10 μM TPP. Reactions were done in the presence of 25 μM NTPs and were incubated for 15 s, 30 s, 45 s, 60 s, 90 s, 2 min, 3 min, 4 min, 5 min, 8 min and 10 min. Chase reactions were done by adding 1 mM NTPs. Transcriptional intermediates are indicated on the right. The transcriptional pause site located between 104 and 117 (shown by a star) was not taken into account due to the very low fraction escaping the pause site. The right panel shows the pause site mapping of *thiB* transcript intermediates. Reaction times were performed for 15 s, 30 s and 45 s.

(b) Sequence of the *thiB* riboswitch showing the predicted P1, anti-P1 and sequestering stems are indicated in green, orange and gray, respectively. The expected region

sequestered by RNAP at the A136 pause site is shown in black. The prediction of paired regions is based on a previous study[4].

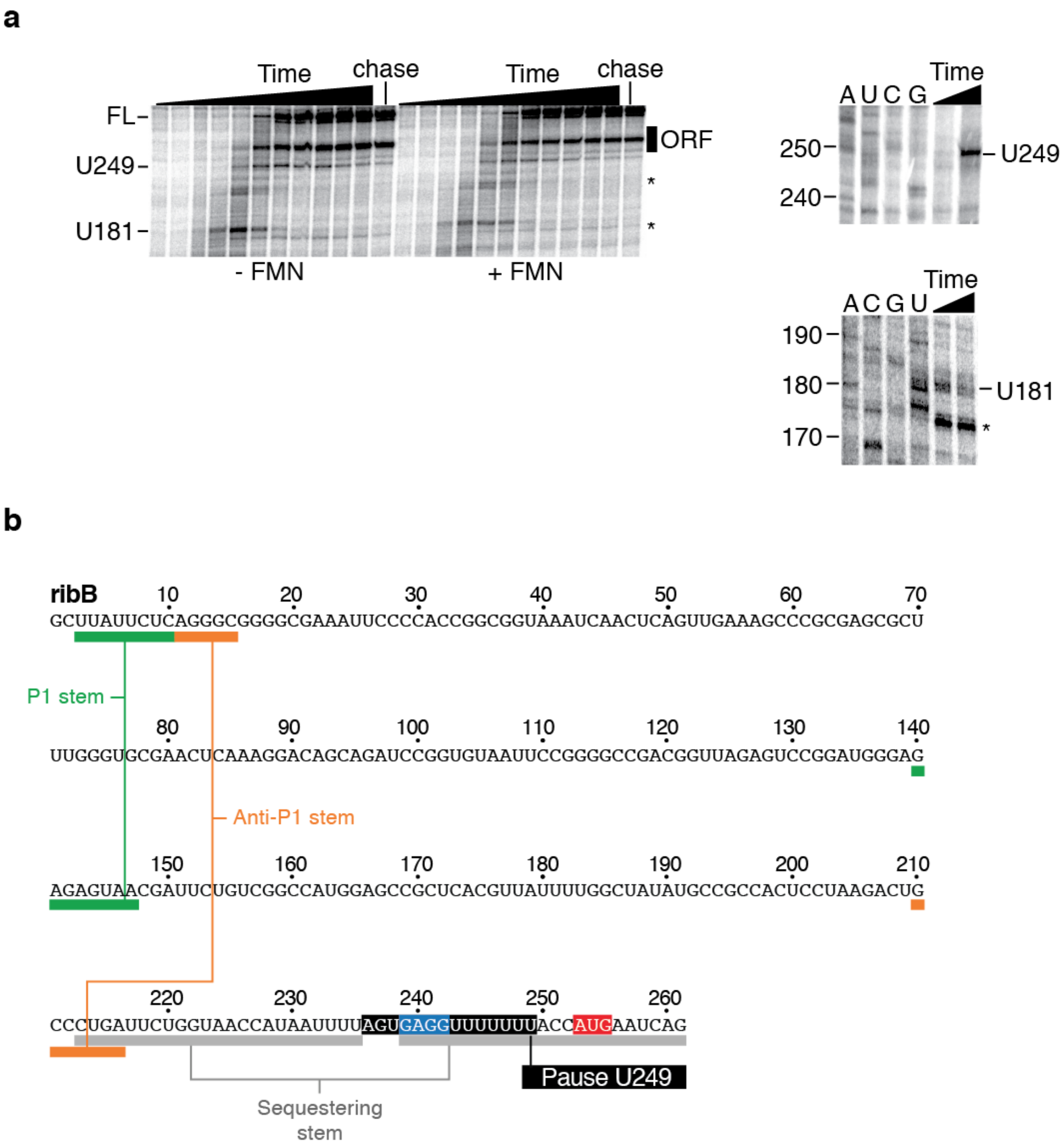

Supplementary Figure 15. Transcriptional pausing of the *ribB* riboswitch.

(a) Transcriptional pausing of the *ribB* riboswitch in the absence or presence of 10 μM FMN. Reactions were done in the presence of 25 μM NTPs and were incubated for 15 s, 30 s, 45 s, 60 s, 90 s, 2 min, 3 min, 4 min, 5 min, 8 min and 10 min. Chase reactions were done by adding 1 mM NTPs. Transcriptional intermediates are indicated on the right. Transcriptional pause sites found downstream in the ORF or exhibiting low efficiency

(shown by a star) were not taken into account. The upper and lower right panels show the pause site mapping of *ribB* transcript intermediates. Due to the large difference in molecular sizes, two different migration times were conducted. Reaction times were performed for 60 s and 90 s (top panel) and 45 s and 60 s (bottom panel).

(b) Sequence of the *ribB* riboswitch showing the predicted P1, anti-P1 and sequestering stems are indicated in green, orange and gray, respectively. The expected region sequestered by RNAP at the U247 pause site is shown in black. The prediction of paired regions is based on a previous study[6].

Fig. 1f

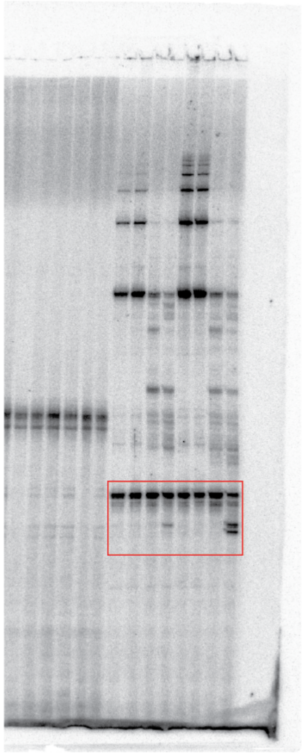

Fig. 2a left panel

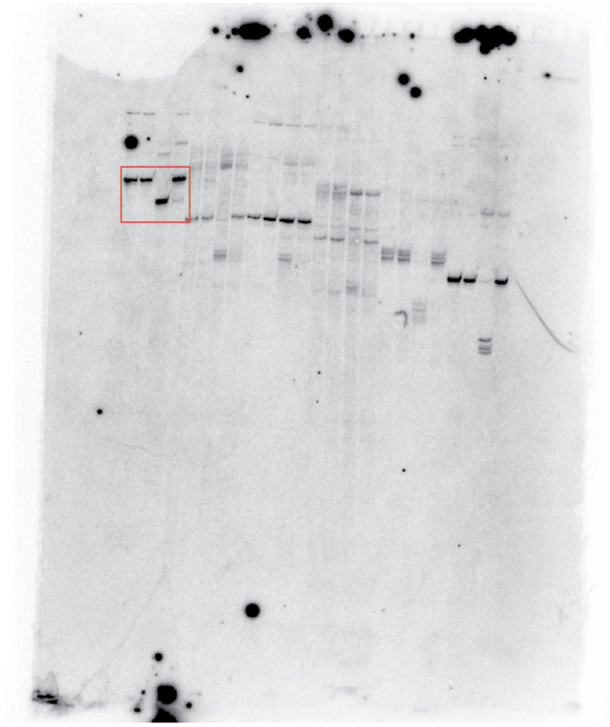

Fig. 1g

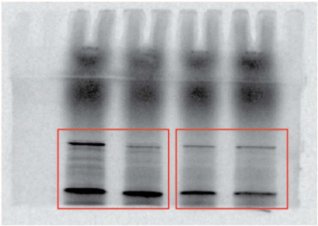

Fig. 2a right panel

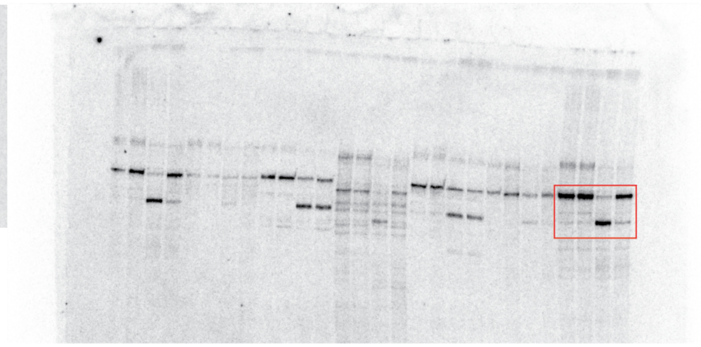

Supplementary Figure 16. Uncropped figures. Boxed areas correspond to images presented in the main text and supplementary figures.

Fig. 2d

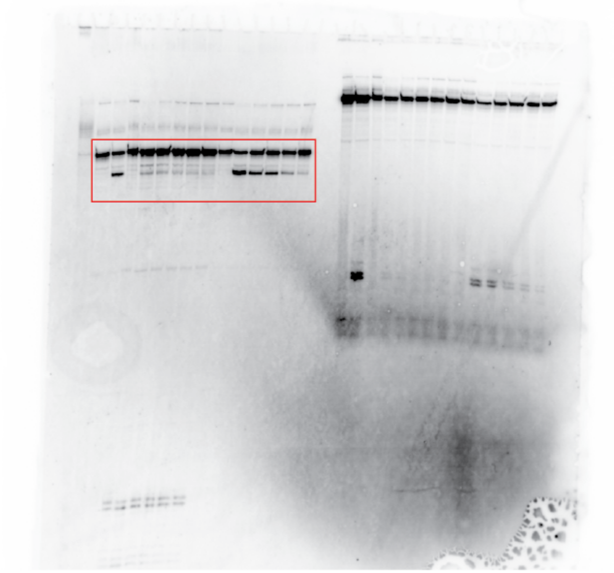

Fig. 2f

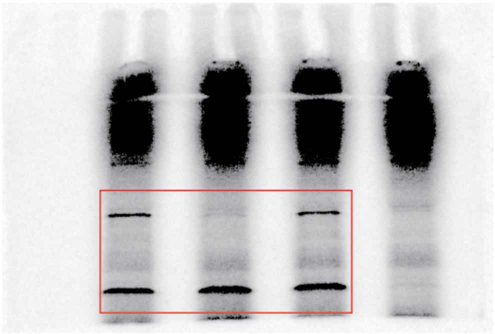

Fig. 3a

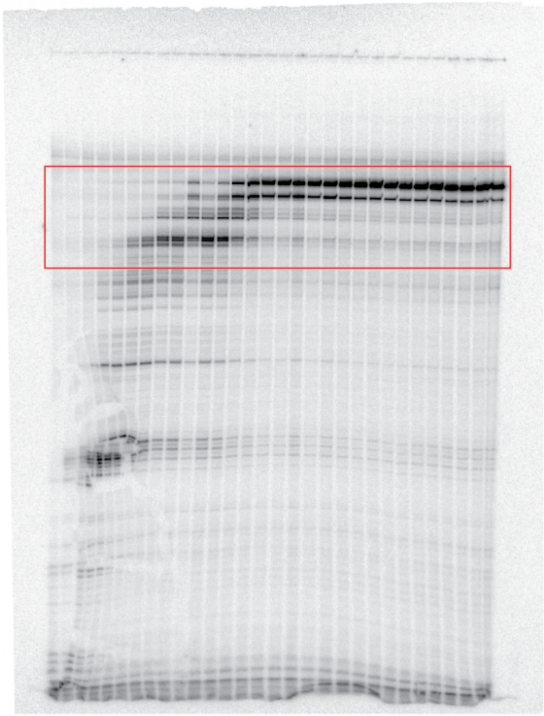

Fig. 3d

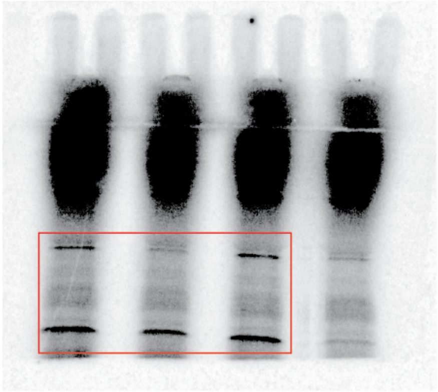

Supplementary Figure 16. Uncropped figures. Boxed areas correspond to images presented in the main text and supplementary figures.

Fig. 4b

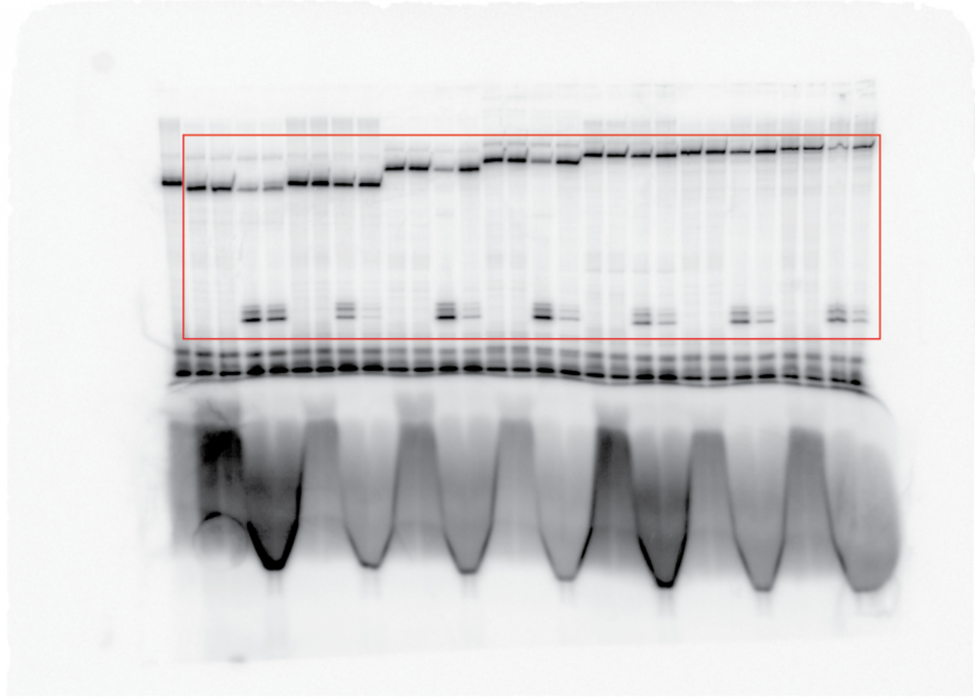

Fig. 4e

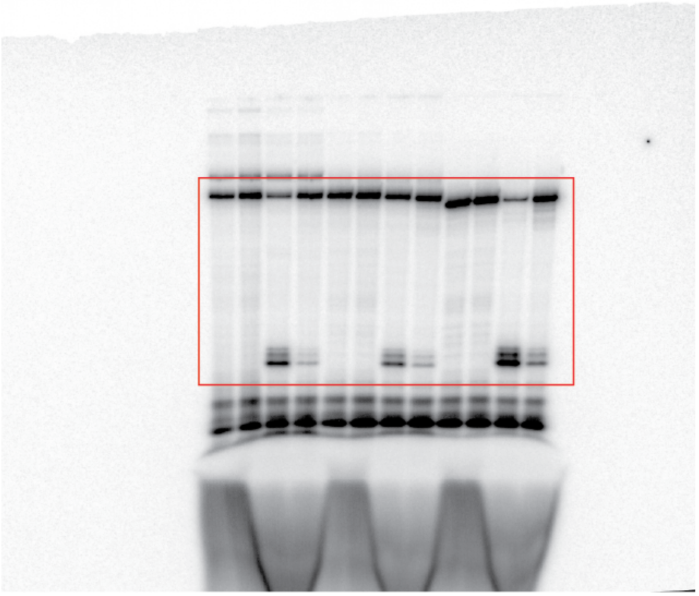

Supplementary Figure 16. Uncropped figures. Boxed areas correspond to images presented in the main text and supplementary figures.

Supplementary Fig. 2a

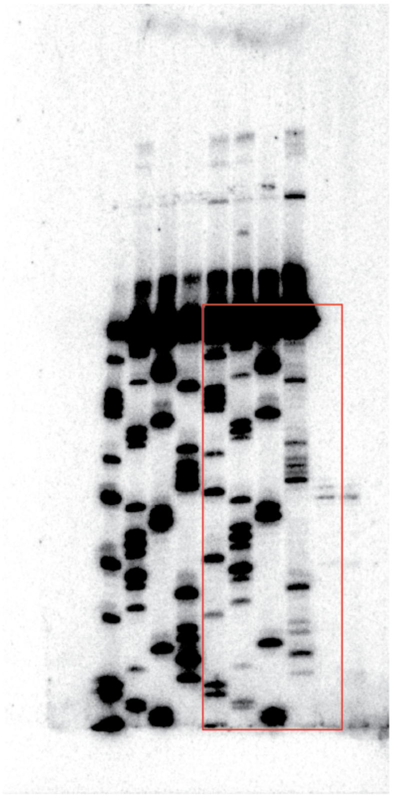

Supplementary Fig. 3a

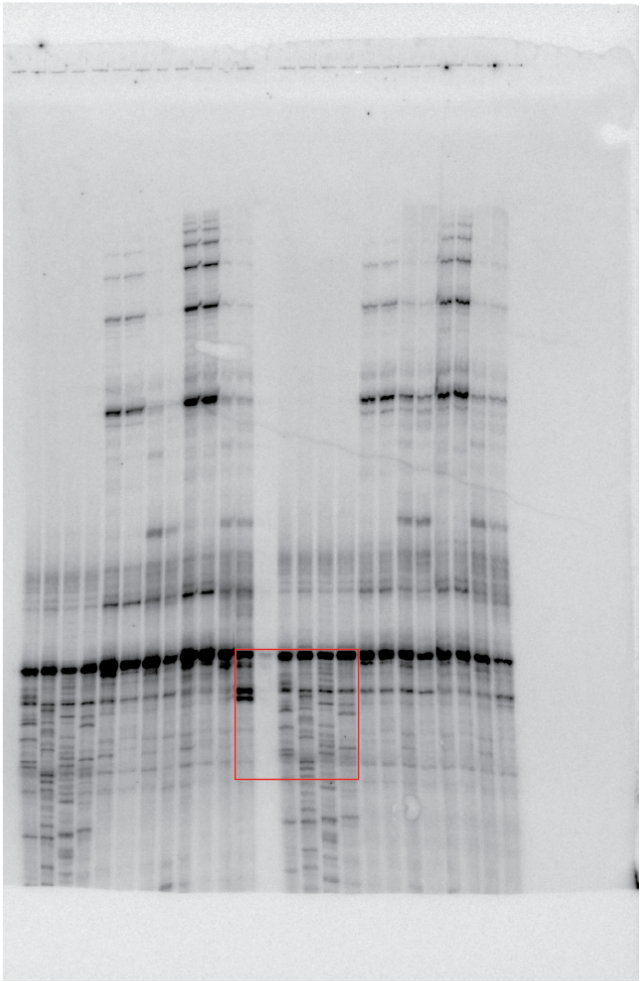

Supplementary Figure 16. Uncropped figures. Boxed areas correspond to images presented in the main text and supplementary figures.

Supplementary Fig. 3c

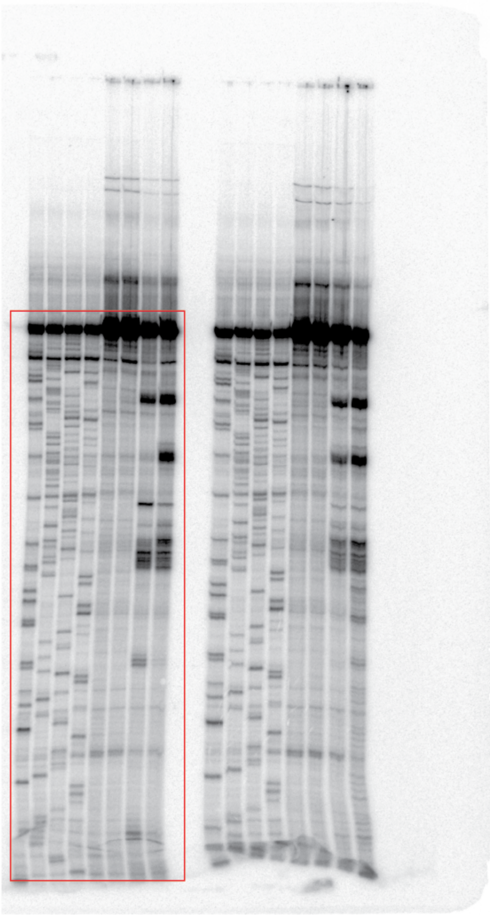

Supplementary Fig. 3d

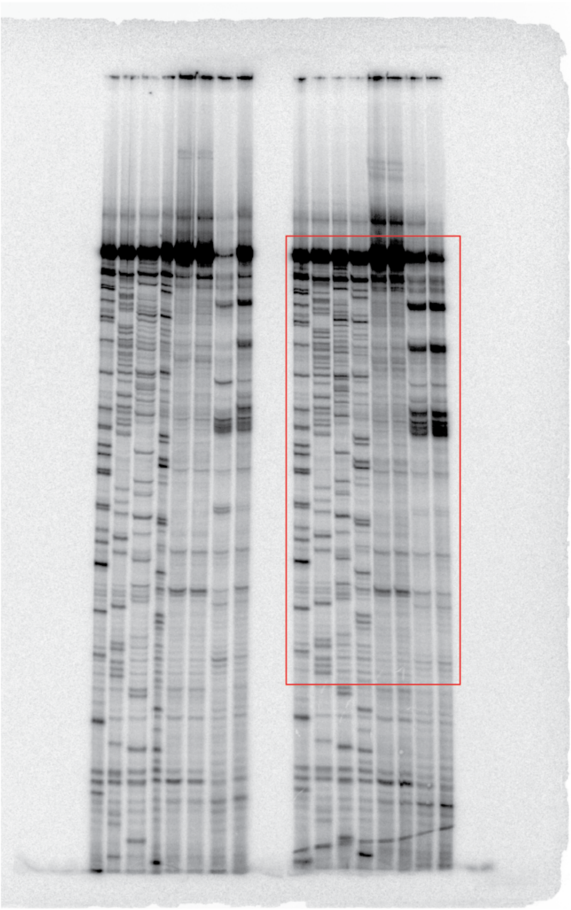

Supplementary Figure 16. Uncropped figures. Boxed areas correspond to images presented in the main text and supplementary figures.

Supplementary Fig. 4a

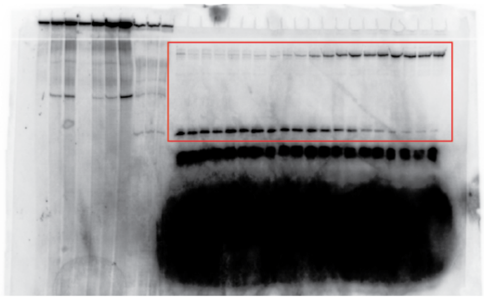

Supplementary Fig. 5b

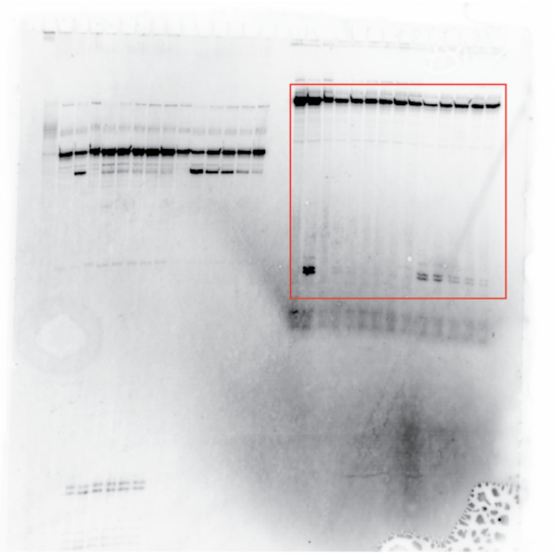

Supplementary Fig. 4b

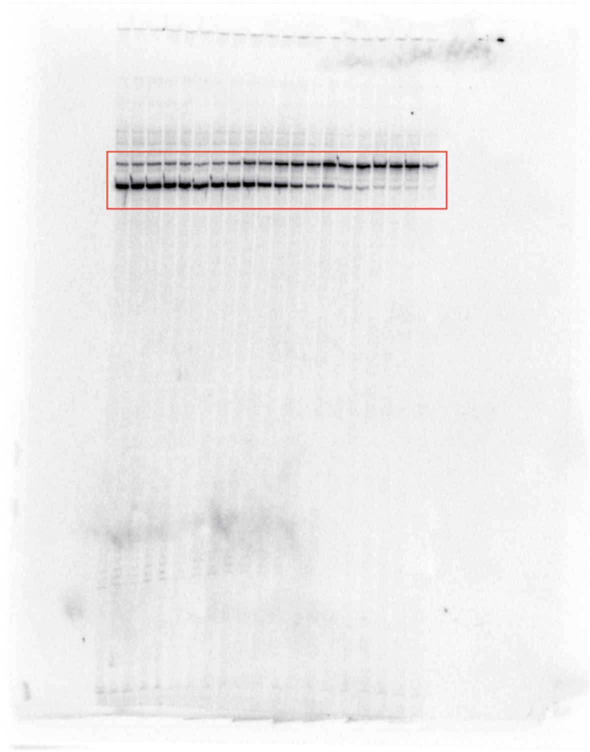

Supplementary Fig. 5d upper panel

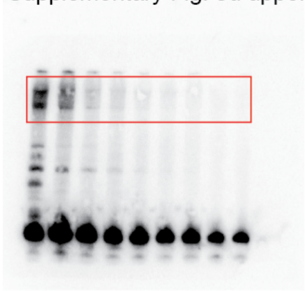

Supplementary Fig. 5d lower panel

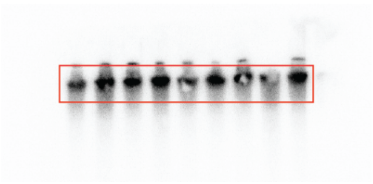

Supplementary Figure 16. Uncropped figures. Boxed areas correspond to images presented in the main text and supplementary figures.

Supplementary Fig. 6a

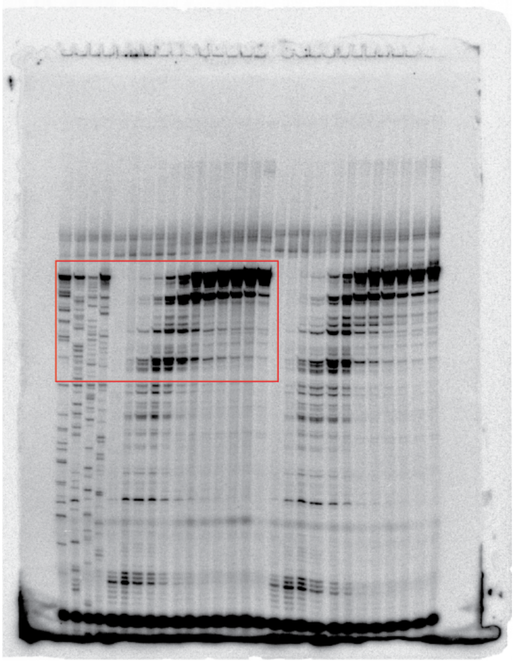

Supplementary Fig. 6b

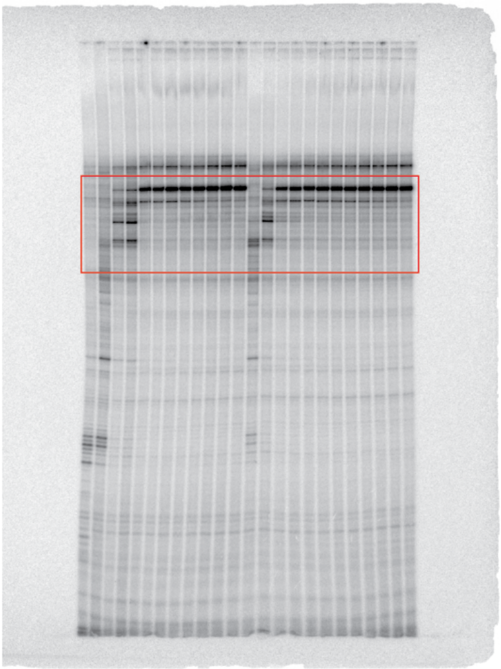

Supplementary Fig. 6c

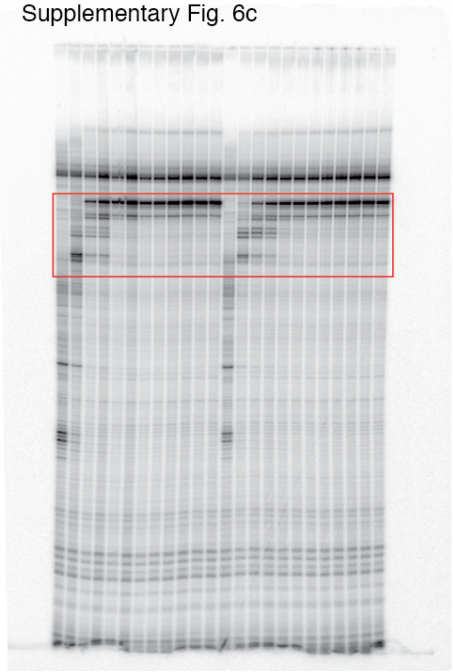

Supplementary Fig. 7a

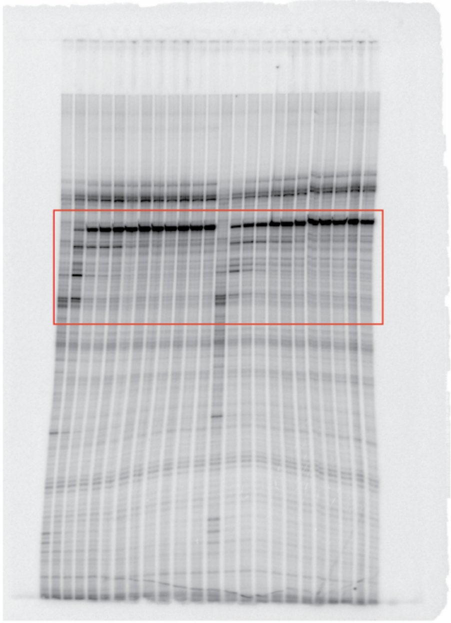

Supplementary Figure 16. Uncropped figures. Boxed areas correspond to images presented in the main text and supplementary figures.

Supplementary Fig. 7b

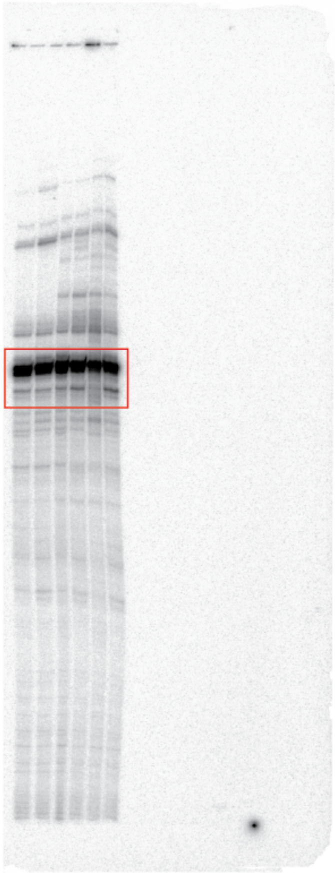

Supplementary Fig. 7c

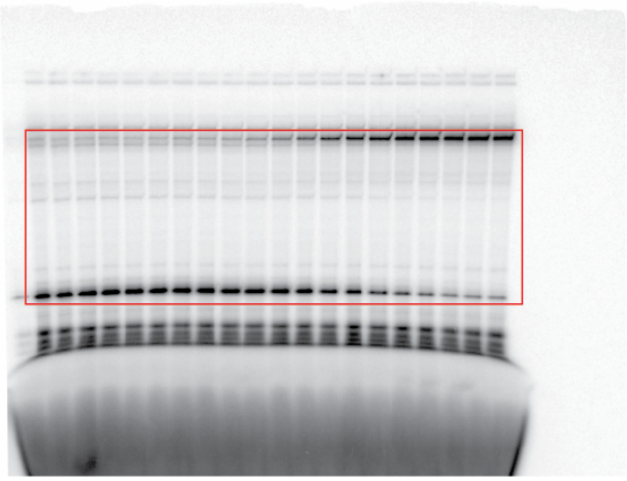

Supplementary Fig. 7d

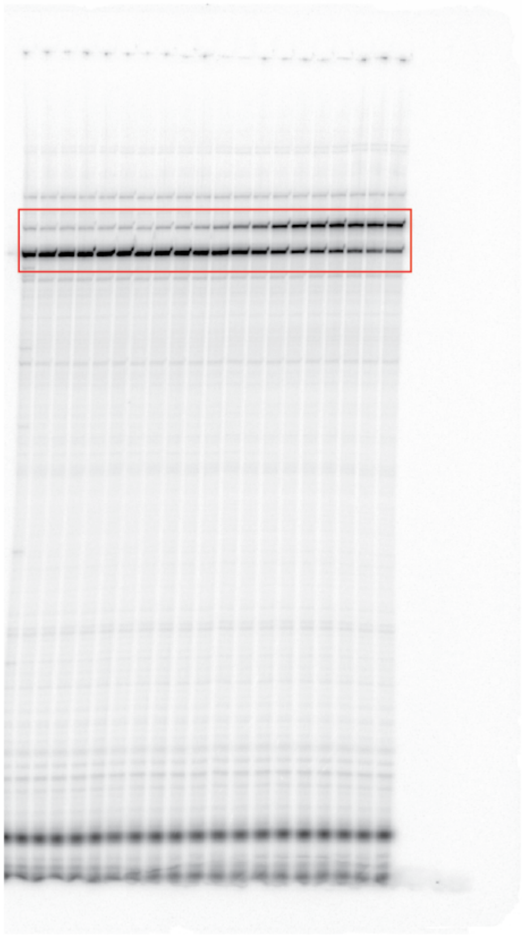

Supplementary Figure 16. Uncropped figures. Boxed areas correspond to images presented in the main text and supplementary figures.

Supplementary Fig. 8a

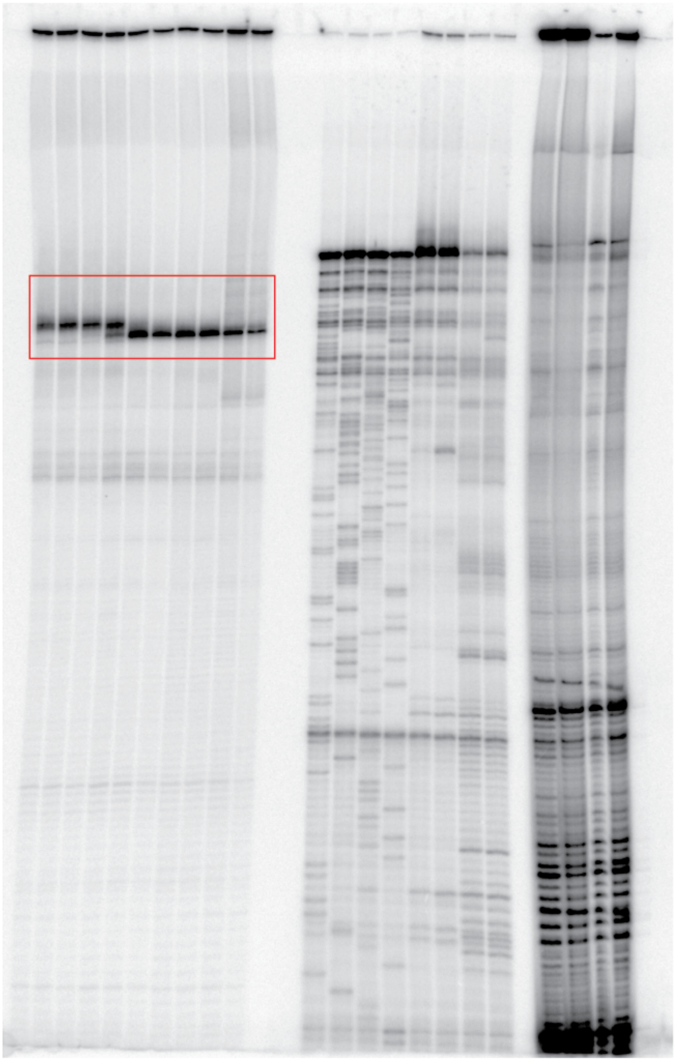

Supplementary Fig. 8b

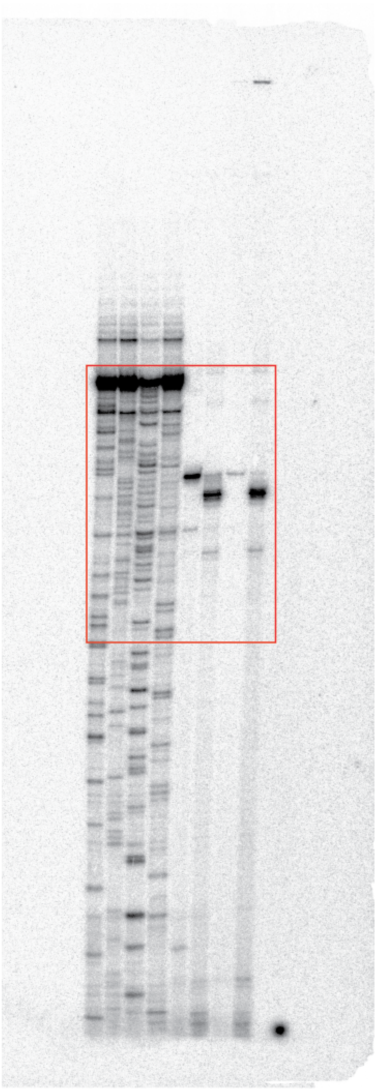

Supplementary Figure 16. Uncropped figures. Boxed areas correspond to images presented in the main text and supplementary figures.

Supplementary Fig. 9a

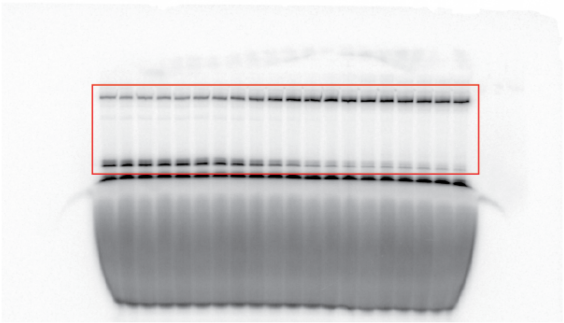

Supplementary Fig. 9b

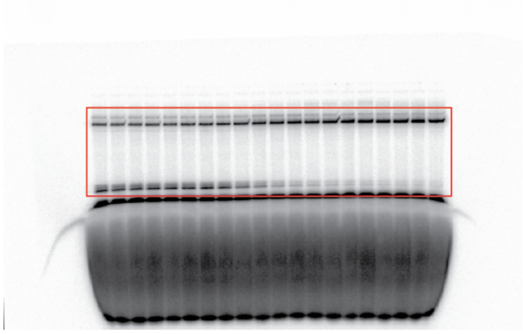

Supplementary Fig. 9c

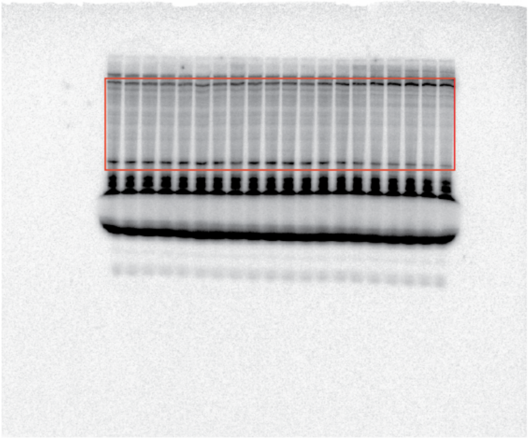

Supplementary Fig. 9d

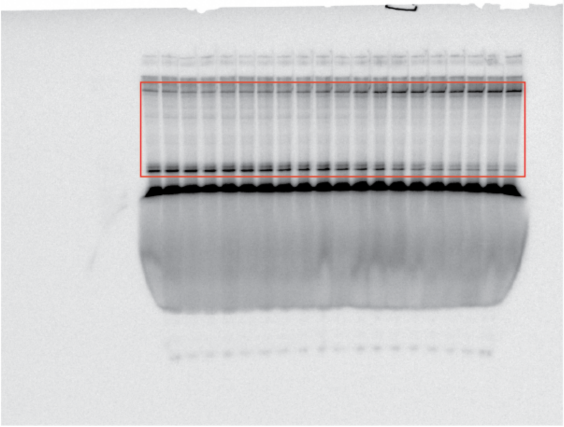

Supplementary Figure 16. Uncropped figures. Boxed areas correspond to images presented in the main text and supplementary figures.

Supplementary Fig. 10a

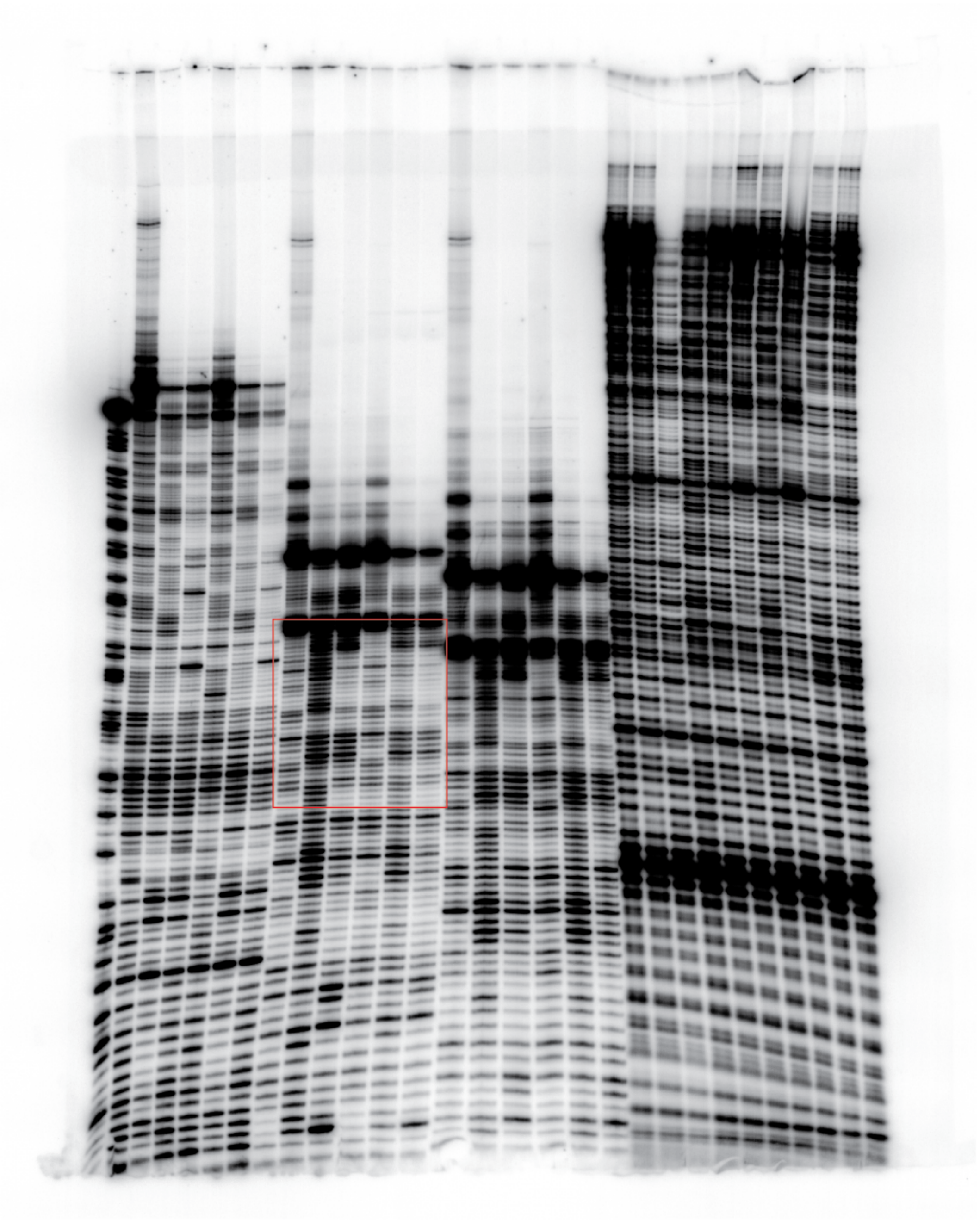

Supplementary Figure 16. Uncropped figures. Boxed areas correspond to images presented in the main text and supplementary figures.

Supplementary Fig. 11a left panel

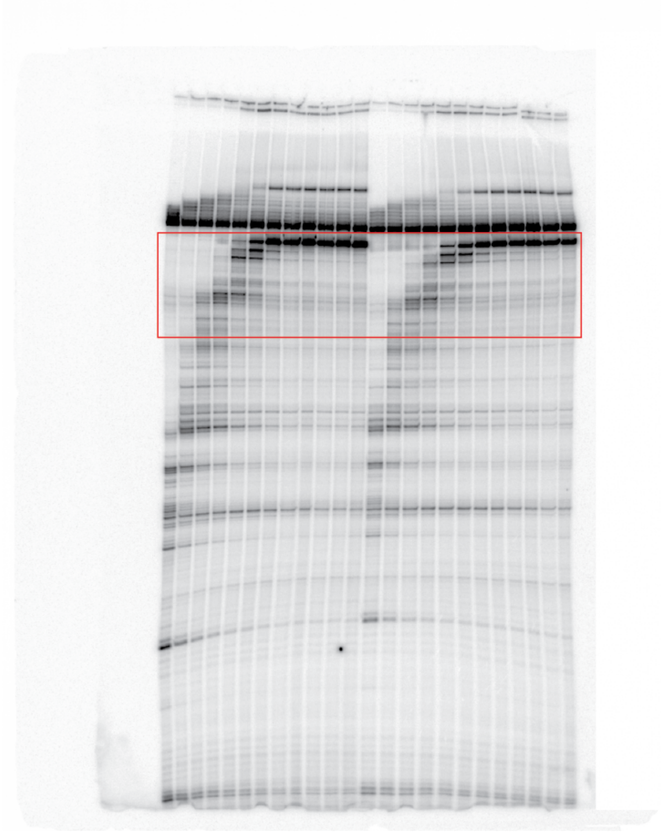

Supplementary Fig. 11a right panel

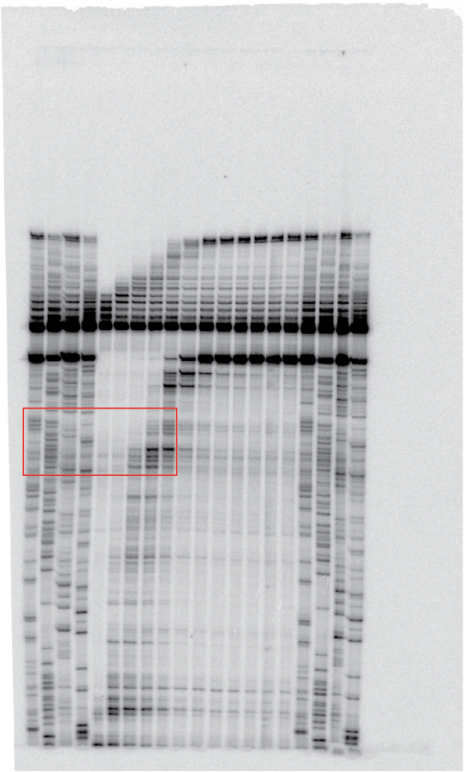

Supplementary Figure 16. Uncropped figures. Boxed areas correspond to images presented in the main text and supplementary figures.

Supplementary Fig. 12a left panel

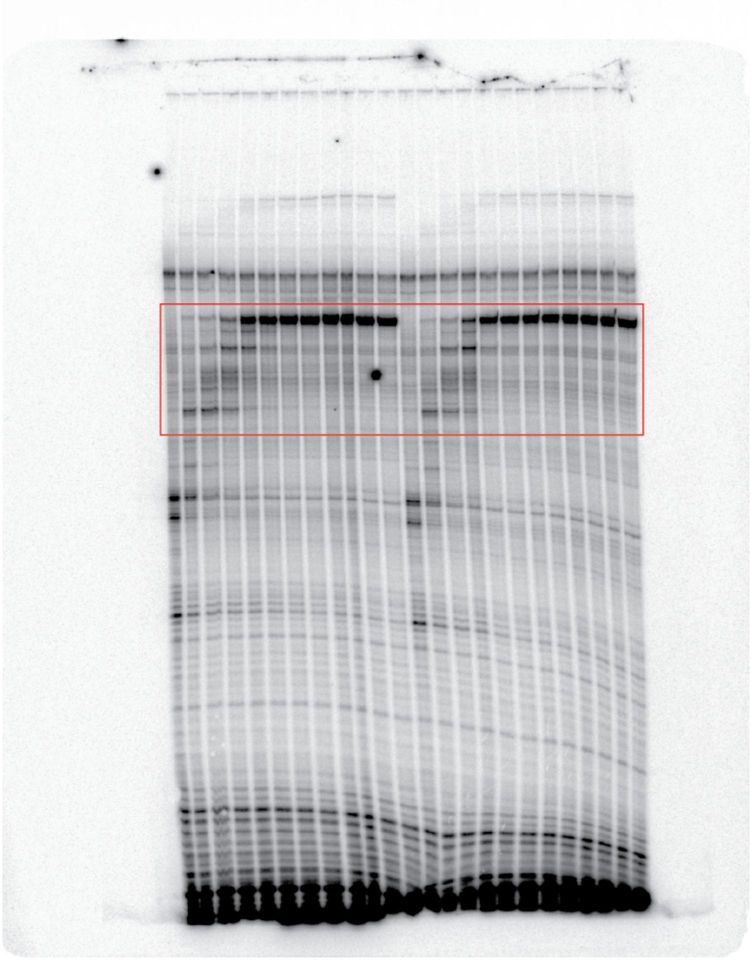

Supplementary Fig. 12a right panel

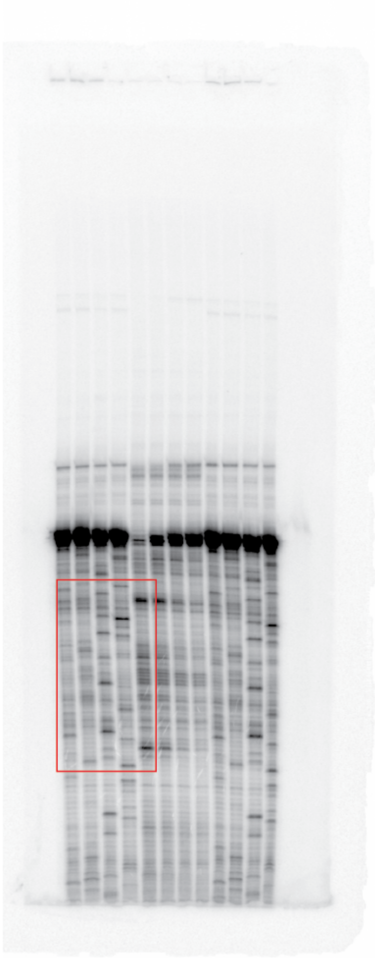

Supplementary Figure 16. Uncropped figures. Boxed areas correspond to images presented in the main text and supplementary figures.

Supplementary Fig. 13a left panel

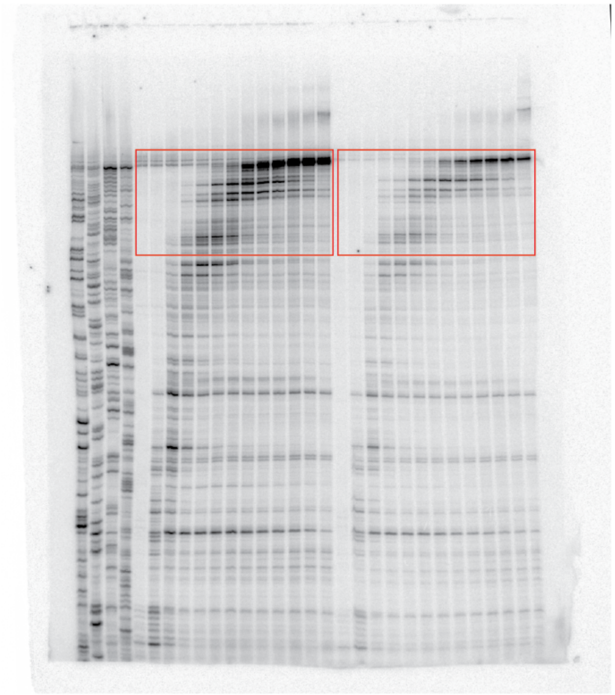

Supplementary Fig. 13a right panel

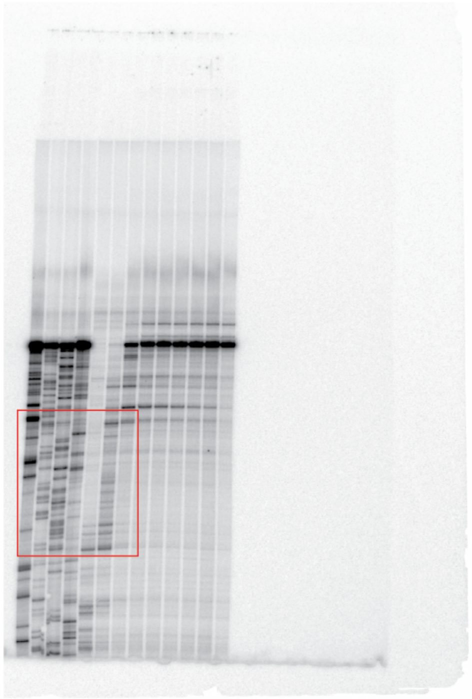

Supplementary Figure 16. Uncropped figures. Boxed areas correspond to images presented in the main text and supplementary figures.

Supplementary Fig. 14a left panel

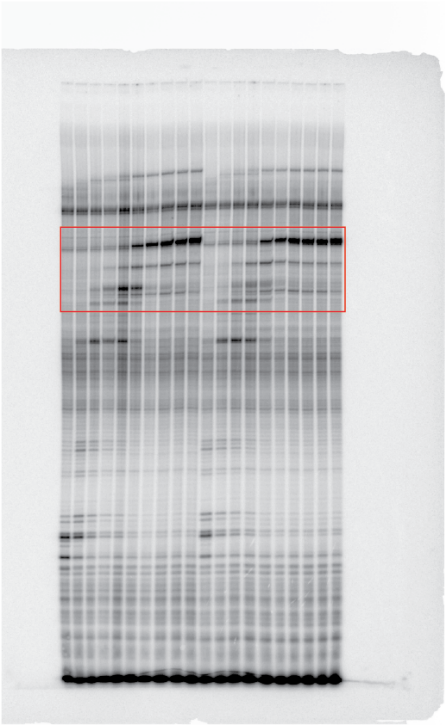

Supplementary Figure 14a right panel

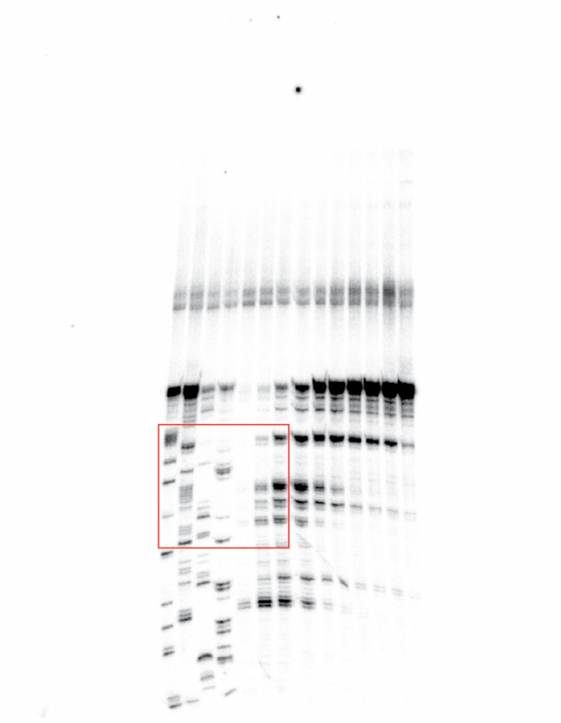

Supplementary Figure 16. Uncropped figures. Boxed areas correspond to images presented in the main text and supplementary figures.

Supplementary Fig. 15a left panel

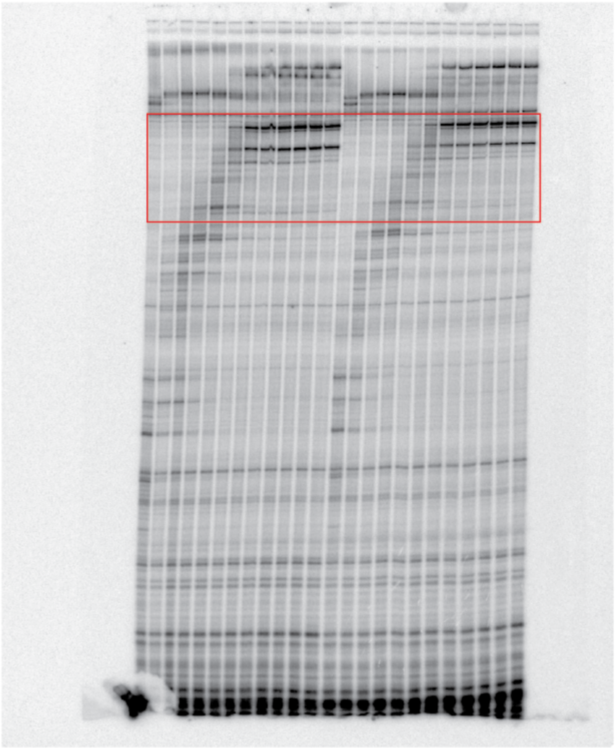

Supplementary Fig. 15a right upper panel

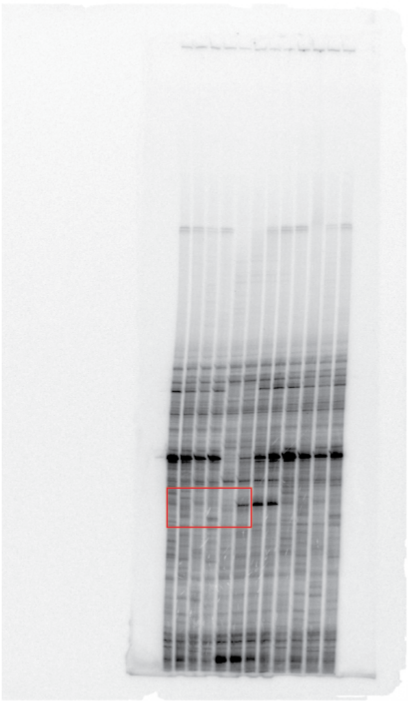

Supplementary Fig. 15a right lower panel

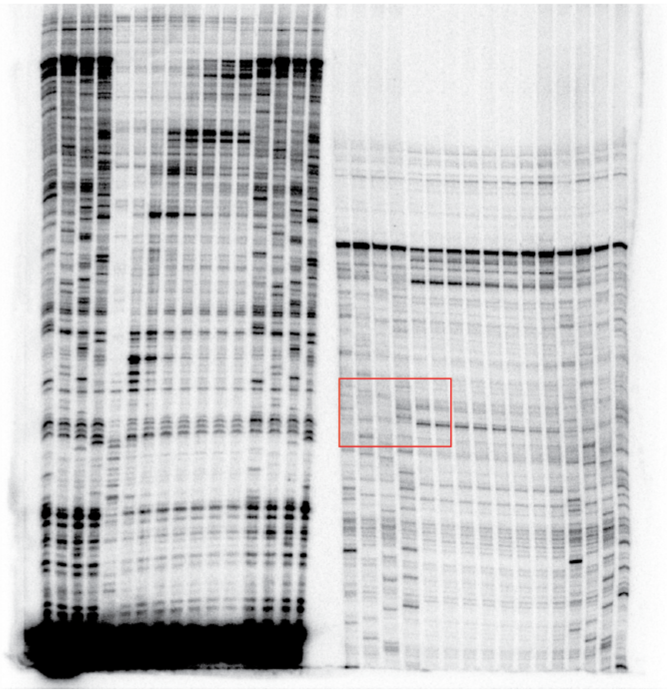

Supplementary Figure 16. Uncropped figures. Boxed areas correspond to images presented in the main text and supplementary figures.

Supplementary Tables

Supplementary Table 1. K_{switch} values determined at various NTP concentrations.

Template	Probes	[NTP]	K_{switch}
Wild type	RBS	1 mM	453 nM ± 13 nM
	RBS	500 μM	222 nM ± 22 nM
	RBS	50 μM	190 nM ± 19 nM
	P1	1 mM	422 nM ± 27 nM
	P1	500 μM	310 nM ± 16 nM
	P1	50 μM	170 nM ± 15 nM

Supplementary Table 2. Half-life values determined in this study.

Templates	TPP	Pause sites	Half-lives
Wild type	-	A138	8 s ± 2 s
	-	C158	9 s ± 3 s
	-	C187	34 s ± 5 s
	+	A138	7 s ± 1 s
	+	C158	7 s ± 1 s
	+	C187	95 s ± 5 s
U160A	-	A138	12 s ± 2 s
	-	C158	9 s ± 5 s
	-	C187	28 s ± 5 s
	+	A138	10 s ± 3 s
	+	C158	8 s ± 3 s
	+	C187	20 s ± 3 s
U186A	-	A138	9 s ± 2 s
	-	C158	12 s ± 1 s
	-	C187	18 s ± 2 s
	+	A138	11 s ± 3 s
	+	C158	12 s ± 2 s
	+	C187	27 s ± 3 s

Supplementary Table 3. Summary of strains or plasmids used in this study.

Strains	Relevant marker	References
EM1055	MG1655 *ΔlacZ* X174	Masse and Gottesman 2002[7]
EM1377	EM1055 *rne131 zce-726*::Tn*10*	Masse *et al*. 2003[8]
PM1205	*lacI*'::P_{BAD}-*cat-sacB-lacZ*, mini l tet^R	Mandin and Gottesman 2009[9]
DAL1	BL21(DE3) F–ompT hsdS(rB- mB-) gal dcm λ (DE3)	Laboratory collection
EM1047	DH5a + pACYC184	Laboratory collection
EM1237	DY330 [W3110 delta-lacU169 gal490 lambda-cI857 delta-(cro-bioA)]	Yu *et al*. 2000[10]
	BL21(DE3) pIA247 (nusG)	Laboratory collection
	BL21(DE3) pET28b-rho	Laboratory collection
TPP7	EM1055::pfr-delta-$ThiC_{11cd}$	This study
TPP8	EM1377::pfr-delta-$ThiC_{11cd}$	This study
TPP1	PM1205 lacI′::P_{BAD}-$ThiC_{10cd}$-LacZ	This study
TPP2	PM1205 lacI′::P_{BAD}-*thiC*$_{10cd}$-*lacZ*	This study
TPP3	PM1205 lacI′::P_{BAD}-$ThiC_{10cd}$-G31C-LacZ	This study
TPP4	PM1205 lacI′::P_{BAD}-$ThiC_{10cd}$-G31C-LacZ	This study
TPP5	PM1205 lacI′::P_{BAD}-$ThiC_{10cd}$-mutOFF-LacZ	This study
TPP6	PM1205 lacI′::P_{BAD}-*thiC*$_{10cd}$-mutOFF-*lacZ*	This study
TPP9	PM1205 lacI′::P_{BAD}-$ThiC_{10cd}$-U186A-LacZ	This study
TPP10	PM1205 lacI′::P_{BAD}-*thiC*$_{10cd}$-U186A-*lacZ*	This study

Supplementary Table 4. Summary of *lacZ* fusions used in this study.

Strains	Constructs	Oligonucleotides
TPP1	ThiC$_{10cd}$	AD17-AD18 (genomic DNA)
TPP2	*thiC*$_{10cd}$	AD17-AD13 (genomic DNA)
TPP3	ThiC$_{10cd}$-G31C	PCR1: AD17-SR-G31C(2) (genomic DNA)
		PCR2: SR-G31C(3)-AD18 (genomic DNA)
		PCR3: AD17-AD18 (PCR1-2)
TPP4	*thiC*$_{10cd}$-G31C	PCR1: AD17-SR-G31C(2) (genomic DNA)
		PCR2: SR-G31C(3)-AD13 (genomic DNA)
		PCR3: AD17-AD13 (PCR1-2)
TPP7	ThiC$_{10cd}$-mutOFF	2174AC-AD18(genomic DNA)
TPP8	*thiC*$_{10cd}$-mutOFF	2174AC-AD13 (genomic DNA)
TPP11	ThiC$_{10cd}$-U186A	AD17-1377AC (genomic DNA)
TPP12	*thiC*$_{10cd}$-U186A	AD17-1378AC (genomic DNA)
**Cloning in plasmid pfr-delta**		
TPP9-10	pfr-delta-*thiC*$_{11cd}$	PCR1: 1429AC-473AC (genomic DNA)
		PCR2: 474AC-473AC (PCR1)

Supplementary Table 5. Summary of oligonucleotides used in this study.

Oligonucleotides	Sequences 5'-3'
5AL	GGGCACCCCAGGCTTTACACTTTATGCTTCCGGCTCGTATAATGTGT GGGCCGGTCCTGTGAGTTAATAG
275AL	GGGCACCCCAGGCTTTACACTTTATGCTTCCGGCTCGTATAATGTGT GGGTTCTCAACGGGGTGCCAC
473AC	TCGAATTCCCGGGTTGTTCGCGGCGGGTCAGTTTTGTTGCAGA
474AC	TGGACCGAATTCGGGCACCCCAGGCTTTACACTTTAT
510AC	GCCACGACGGATGAAGCAAGAGACG
511AC	CCGTCGTGGCACAAGCCACGTCCTTAACTT
663AC	TGAAGCAAGAGACGATCGCCGCAG
664AC	GTGGCTTGTCAGACGACGGATGAAGC
721AC	AAAAAGTTAAGGTCGTGGCTTGTCAGACG
722AC	CGTCTGACAAGCCACGACCTTAACTTTTT
765LB	CGGGCTGGTATCCTGTG
827AC	TTGTTCGCGGCGGGTCAGTTTTGTTGCAGTCATAGCTCATTC
918AC	CAACAGCAGGGGCAGACATTTTTTTAACAC
989MG	GGGCACCCCAGGCTTTACACTTTATGCTTCCGGCTCGTATAATGTGT GGGGGCTTAAGTATAAGGAGGAAAAAATATG
1041AC	CGCCCGCGTCAAACATCCTGCTTGA
1168AC	GGGCACCCCAGGCTTTACACTTTATGCTTCCGGCTCGTATAATGTGT GGGTACTACCTGCGCTAGCGCA
1178AC	GTTTTGTTGCAGTCATAGCTCATTCCA
1179AC	TGGAATGAGCTATGACTGCAACAAAAC
1194AC	GCACGTTGGCATCAGAAAGCA
1231MG	AAACCCCTCCGTTTAGAGAGGGGTTATGCTAGTTAGGCGCGTAAAA ATGCGCTC
1232MG	AAACCCCTCCGTTTAGAGAGGGGTTATGCTAGTTAGAACGCTTCCC AGGTAAGATCTTC
1372AC	GGGCACCCCAGGCTTTACACTTTATGCTTCCGGCTCGTATAATGTGT GGCTGCGATTTATCATCGCAACCAAAC
1377AC	AACGCCAGGGTTTTCCCAGTCACGACGTTGTAAAACGACTTCGCGG CGGGTCAGTTTTGTTGCAGTCAT
1378AC	GTGTGATAAAGAAAGTTAAAATGCCGGATCTTCGCGGCGGGTCAGT TTTGTTGCAGTCAT
1429AC	GGGCACCCCAGGCTTTACACTTTATGCTTCCGGCTCGTATAATGTGT GGTAATTTCTTGTCGGAG
1489FPJ	5'biotin-GCGGGTCAGTTTTGTTGCAGA
1494FPJ	GACATAGCTCATTCCAAAAAG
1495FPJ	GACATAGCTCATTCCAAAAAGTTAAG
1697AC	5'biotin-ATGCGATAGCAGATTAACTCTTGTTCCC
1698AC	5'biotin-CAGGGGCGATGCGATAGCAGATTAA
1704AC	5'biotin-GTTGTTCGCGGCGGGTCAGTTTTGT
1710AC	TTGTTCGCGGCGGGTCAGTTTTGTTGCAGA
1789FPJ	TGCGATAGCAGATTAACTCTTGT
1790FPJ	GGGCGATGCGATAGCAGATTAAC

2019AC	ACCAAAAGAGGAAAGTAGCGTCTGATTCAT
2020AC	TTGCGCTGAACCCAGCAGGTCGACT
2174AC	ACCTGACGCTTTTTATCGCAACTCTCTACTGTTTCTCCATTTAACTCT TGTCGGAGTGCCTTAACTGG
2247AC	5'biotin-GACGACGGATGAAGCAAGAGACG
2248AC	5'biotin-GTTAAGGACGTGGCTTGTCAGACG
2249AC	5'biotin-TTTGTTGCAGACATAGCTCATTCC
AD5	CGGATCCCGAATAAACGGTCTC
AD13	GTGTGATAAAGAAAGTTAAAATGCCGGATCTTCGCGGCGGGTCAGT TTTGTTGCAGACAT
AD17	ACCTGACGCTTTTTATCGCAACTCTCTACTGTTTCTCCATTAATTTCT TGTCGGAGTGCC
AD18	TAACGCCAGGGTTTTCCCAGTCACGACGTTGTAAAACGACTTCGCG GCGGGTCAGTTTTGTTGCAGACAT
AD113	CCGGCGTGATGATGCCCTGGCGGGCGTAGTG
Oligo-stop	TAACGCCAGGGTTTTCCCAGTCACGACGTTGTAAAACGACCATAGC TGTTTCCTGTGTGATAAAGAAAGTTAAAATGCCGGATC
P1	GCACTCCGAC
RBS	GCTCATTCCA
RibBL1	TTCCCTGTACTGATAGGTGTTG
SR-G31C(2)	ATCCCGAATAAACGGTCTGAGCCAGTT
SR-G31C(3)	AACTGGCTCAGACCGTTTATTCGGGAT

Supplementary Table 6. PCR constructs used for *in vitro* RNA synthesis.

Constructions	Oligonucleotides
***In vitro* transcription assays**	
pLacUV5-btuB-315	5AL-765LB (genomic DNA)
pLacUV5-lysC-406	1168AC-1194AC (genomic DNA)
pLacUV5-thiB-158	275AL-918AC (genomic DNA)
pLacUV5-thiM-180	1372AC-2020AC (genomic DNA)
pNat-ribB-282	ribBL1-2019AC (genomic DNA)
pNAT-thiC-138	1041AC-663AC (genomic DNA)
pNAT-thiC-143	1041AC-816AC (genomic DNA)
pNAT-thiC-158	1041AC-664AC (genomic DNA)
pNat-thiC-215	1041AC-1710AC (genomic DNA)
pNat-thiC-215-U186A	1041AC-827AC (genomic DNA)
pNat-thiC-215-U160A	PCR1: 1041AC-721AC (genomic DNA)
	PCR2: 722AC-1710AC (genomic DNA)
	PCR3: 1041AC-1710AC (PCR1-2)
***In vitro* transcription-translation assays**	
pLacUV5-lacZ	98MG-1231MG (genomic DNA)
pLacUV5-thiC$_{315cd}$	1429AC-1232MG (genomic DNA)
pLacUV5-thiC$_{315cd}$-G31C	PCR1: 1429AC-SR-G31C(2) (genomic DNA)
	PCR2: SR-G31C(3)-1232MG (genomic DNA)
	PCR3: 1429AC-1232MG (PCR1-2)
pLacUV5-thiC$_{315cd}$-U186A	PCR1: 1429AC-1178AC (genomic DNA)
	PCR2: 1179AC-1232MG (genomic DNA)
	PCR3: 1429AC-1232MG (PCR1-2)
**Transcription elongation complexes**	
pNat-thiC-EC100	1041AC-1697AC (genomic DNA)
pNat-thiC-EC108	1041AC-1698AC (genomic DNA)
pNat-thiC-EC138	1041AC-2247AC (genomic DNA)
pNat-thiC-EC158	1041AC-2248AC (genomic DNA)
pNat-thiC-EC187	1041AC-2249AC (genomic DNA)
pNat-thiC-EC187-Anti-P1 mutant	PCR1: 1041AC-510AC (genomic DNA)
	PCR2: 511AC-2249AC (genomic DNA)
	PCR3: 1041AC-2249AC (PCR1-2)
pNat-thiC-EC197	1041AC-1489FPJ (genomic DNA)
pNat-thiC-EC207	1041AC-1704AC (genomic DNA)

Supplementary References

1. Weinberg, Z. & Breaker, R. R. R2R--software to speed the depiction of aesthetic consensus RNA secondary structures. *BMC Bioinformatics* **12,** 3 (2011).
2. Caron, M. P. *et al.* Dual-acting riboswitch control of translation initiation and mRNA decay. *Proc Natl Acad Sci U S A* **109,** E3444-53 (2012).
3. Nahvi, A. *et al.* Genetic control by a metabolite binding mRNA. *Chem Biol* **9,** 1043 (2002).
4. Winkler, W., Nahvi, A. & Breaker, R. R. Thiamine derivatives bind messenger RNAs directly to regulate bacterial gene expression. *Nature* **419,** 952–956 (2002).
5. Sudarsan, N., Wickiser, J. K., Nakamura, S., Ebert, M. S. & Breaker, R. R. An mRNA structure in bacteria that controls gene expression by binding lysine. *Genes Dev* **17,** 2688–2697 (2003).
6. Winkler, W. C., Cohen-Chalamish, S. & Breaker, R. R. An mRNA structure that controls gene expression by binding FMN. *Proc Natl Acad Sci U S A* **99,** 15908–15913 (2002).
7. Masse, E. & Gottesman, S. A small RNA regulates the expression of genes involved in iron metabolism in Escherichia coli. *Proc Natl Acad Sci U S A* **99,** 4620–4625 (2002).
8. Masse, E., Escorcia, F. E. & Gottesman, S. Coupled degradation of a small regulatory RNA and its mRNA targets in Escherichia coli. *Genes Dev* **17,** 2374–2383 (2003).
9. Mandin, P. & Gottesman, S. A genetic approach for finding small RNAs regulators of genes of interest identifies RybC as regulating the DpiA/DpiB two-component system. *Mol Microbiol* **72,** 551–565 (2009).
10. Yu, D. *et al.* An efficient recombination system for chromosome engineering in Escherichia coli. *Proc Natl Acad Sci U S A* **97,** 5978–5983 (2000).
